# Asynchronous changes of hydrogen sulfide and its generating enzymes in most tissues with the aging process

**DOI:** 10.1042/BSR20240320

**Published:** 2024-10-11

**Authors:** Kaichuan He, Bo Tan, Ao Lu, Lu Bai, Chengqing Song, Yuxin Miao, Biyu Liu, Qian Chen, Xu Teng, Jing Dai, Yuming Wu

**Affiliations:** 1 Department of Physiology, Hebei Medical University, Hebei 050017, China; 2 Center for Clinical Medical Research, Hebei Genral Hospital, Hebei 050051, China; 3 Hebei Key Laboratory of Metabolic Diseases, Hebei Genral Hospital, Hebei 050051, China; 4 Clinical Pharmacokinetic Laboratory, Shuguang Hospital Affiliated to Shanghai University of Traditional Chinese Medicine, Shanghai 201203, China; 5 Department of Clinical Diagnostics, Hebei Medical University, Hebei 050017, China; 6 Hebei Collaborative Innovation Center for Cardio-Cerebrovascular Disease, Hebei 050017, China; 7 The Key Laboratory of Neural and Vascular Biology, Ministry of Education, Shijiazhuang 050017, China; 8 Hebei Key Laboratory of Cardiovascular Homeostasis and Aging, Shijiazhuang 050017, China

**Keywords:** 3-MST, Aging, CBS, CSE, H2S

## Abstract

Aging is an inevitable and irreversible biological process that gradually heightens the risks of various diseases and death. As a newly discovered endogenous gasotransmitter, hydrogen sulfide (H_2_S) has been identified to exert multiple beneficial impacts on the regulation of aging and age-related pathologies. This study was aimed at systematically exploring the relationship between asynchronous aging processes and H_2_S concentrations in various tissues of aging mice. Samples of plasma and 13 tissues were collected from four cross-sectional age groups (3, 6, 12 and 18 months of age) covering the lifespan of male C57BL/6J mice. The H_2_S concentration was quantified by a reported liquid chromatography-tandem mass spectrometry (LC-MS/MS) method with monobromobimane derivatization. Additionally, the expressions of cystathionine γ-lyase (CSE), cystathionine β-synthase and 3-mercaptopyruvate sulfurtransferase, in those tissues were analyzed by Western blotting. We discovered that the H_2_S concentrations decreased asynchronously with the aging process in plasma, heart, liver, kidney, spleen, subcutaneous fat and brown fat and increased in brain and lung. At least one of the three H_2_S-generating enzymes expressions was compensatorily up-regulated with the aging process in most tissues, among which the up-regulation of CSE was the most prominent.

## Introduction

Hydrogen sulfide (H_2_S), a colorless and flammable gas with a distinctive odor reminiscent of rotten eggs, has conventionally been regarded as a toxic environmental pollutant on account of its inhibitory impact on mitochondrial respiration [1]. However, similar to carbon monoxide (CO) and nitric oxide (NO), the initial negative perception of H_2_S has undergone a dramatic shift since the landmark discovery that H_2_S can act as an endogenous gasotransmitter under normal conditions by Abe and Kimura in 1996 [2]. Since then, it has become increasingly evident that H_2_S is endogenously synthesized in diverse mammalian tissues from L-cysteine and homocysteine via the transulfuration pathway by three principal enzymes: cystathionine γ-lyase (CSE), cystathionine β-synthase (CBS) and 3-mercaptopyruvate sulfurtransferase (3-MST), which are widely expressed with distinct tissue distribution patterns. Among them, CSE is abundantly expressed in the cardiovascular system [3]. Whereas CBS is predominantly situated in the central nervous system [4]. 3-MST is prevalently present not only in mitochondria but also in the cytosol of the majority of tissues [5]. The accumulated evidence suggests that endogenous production of H_2_S is intricately and precisely regulated to maintain physiological homeostasis in various systems of the mammalian body by interacting with and/or modifying target membrane ion channels, proteins, enzymes and transcription factors through four main routes. (1) The first route involves the quenching of reactive oxygen species (ROS) [6], (2) coordination to metal centers [7], (3) interaction with disulfide bonds [8] and (4) S-persulfidation on protein cysteine residues [9]. On the other hand, studies have revealed that the lack of adequate levels of H_2_S could throw cell homeostasis off balance and contribute to various disease including pathologies related to aging [10]. It was unfolded that children had higher plasma levels of H_2_S than adults [11] and plasma H_2_S levels were decreased with age in adults [12]. Preclinical studies have revealed that exogenous H_2_S prolonged the lifespan of *Caenorhabditis elegans* [13] and prevented aging and age-related pathologies [14]. Considering the asynchronous aging process [15] and differences in H_2_S concentrations among various tissues [16], understanding how tissue-specific changes in H_2_S concentrations and its generating enzymes as aging progresses is essential, which is exactly the purpose of the present study.

## Materials and methods

### Animals and treatments

Male C57BL/6J mice were obtained from Liaoning Changsheng Biotechnology Co., Ltd (Liaoning, China), and the study was conducted at Hebei Medical University. The mice were kept in a facility with a 12-h light and dark cycle, humidity maintained at 60% and temperature ranging from 22 to 24°C. They were fed a standard rat chow and provided with tap water ad libitum until they reached 3, 6, 12 and 18 months of age, respectively.

At the experimental endpoint, mice were anesthetized using 1% isoflurane and subsequently killed during anesthesia by intraperitoneal injection of a lethal dose of thiopental (200 mg/kg) followed by cervical dislocation. Blood was collected via cardiac puncture, and plasma was then separated after centrifugation at 3500 rpm for 10 min. The tissues were dissected in the following sequence: brain, cerebellum, spleen, heart, aorta, lung, liver, kidney, colon, skeletal muscle (gastrocnemius), skin (dorsal), subcutaneous fat (inguinal pad) and brown fat (interscapular pad). Plasma and 13 tissues were snap-frozen in liquid nitrogen immediately and stored at −80°C until further assay.

All our animal experimental protocols were executed in accordance with the Guide for the Care and Use of Laboratory Animals of the National Institutes of Health (NIH) of the United States and sanctioned by the Ethics Committee for Laboratory Animals Care and Use of Hebei Medical University.

### Measurement of H_2_S levels

The H_2_S levels in plasma and tissues were measured according to previously described methods [17]. Tissues were homogenized in cold Tris-HCl (100 mmol/L, pH 8.5) followed by centrifugation at 12000 rpm for 20 min at 4°C. The supernatants were separated for measurement and protein concentrations were quantified by BCA assay. Thirty microliters of plasma, erythrocyte or supernatant were mixed with 10 μl of 0.1% ammonia and 80 μl of monobromobimane (MBB, Sigma-Aldrich Ltd., St. Louis., U.S.A.) with shaking for 1 h at room temperature for derivatization of sulfide. MBB reacts with sulfide to produce sulfide-dibimane, which can be separated by gradient elution and analyzed by liquid chromatography-tandem mass spectrometry. The reaction was then terminated with 10 μl of 20% formic acid and centrifuged at 15000 ***g*** for 10 min. After determined by using a curve generated with sodium sulfide (0–40 μmol/L) standards, H_2_S levels in plasma and erythrocyte were expressed as μmol/L. H_2_S levels in tissues were divided by the protein concentrations and were expressed as μmol/g of protein.

### Western blot analysis

Frozen tissues were homogenized with ice-cold RIPA lysis buffer. Proteins were extracted and quantified by the BCA reagent. Equal amount of protein samples were separated on 10% SDS-PAGE gels and transferred to polyvinylidene fluoride membranes. The membranes were blocked with 5% non-fat milk at room temperature for 1 h and then incubated with primary antibodies that recognized CSE (1:500, Santa Cruz, U.S.A.), CBS (1:2000, Proteintech, U.S.A.), 3-MST (1:2000, Santa Cruz, U.S.A.) and GAPDH (1:2000, Proteintech, U.S.A.) at 4°C overnight. After incubation with secondary antibody (1:2000, Proteintech, U.S.A.) and extensive washing with TBST, blots were imaged with SuperSignal West Pico Chemiluminescent Substrate (Thermo, Scientific-Pierce, U.S.A.), and the grey values of the bands were quantified by ImageJ software.

### Statistical analysis

Results were expressed as mean ± SEM. Statistical analysis was performed using an SPSS software package, version 13.0 (SPSS, Inc., Chicago, U.S.A.). For a comparison of more than two groups, one-way ANOVA was applied to compare values between multiple groups. The least significant difference test was used for data with homogeneity of variance and the Dunnett’s T3 test was used for data with heterogeneity of variance. *P*<0.05 was considered statistically significant.

## Results

### H_2_S levels and the activities and expressions of its generating enzymes in tissues during aging

**Figure 1:**
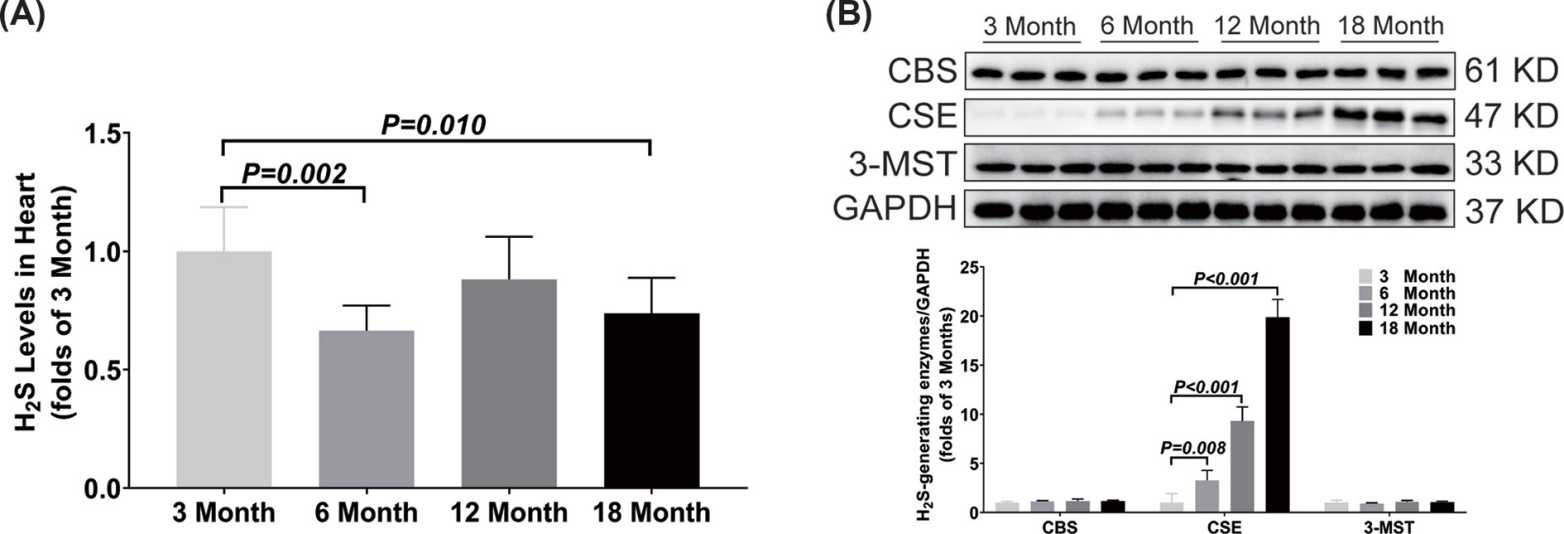
Changes of H_2_S content and H_2_S-generating enzymes expression in heart during aging (**A**) LC-MS/MS was used to detect the content of H_2_S in heart. (**B**) Western blotting images and bar graph comparing the expression levels of CBS, CSE and 3-MST expression levels in heart. Results are means ± SD, *n*=6. A *P* of <0.05 was considered significant.

In heart, the H_2_S concentration was not significantly different between the 3-month-old group and the 12-month-old group, but the 6-month-old group and the 18-month-old group were significantly lower than that in the 3-month-old group (Figure 1A). The CBS and 3-MST expressions were not significantly different, while the CSE expression was gradually up-regulated among the four different age groups (Figure 1B).

**Figure 2:**
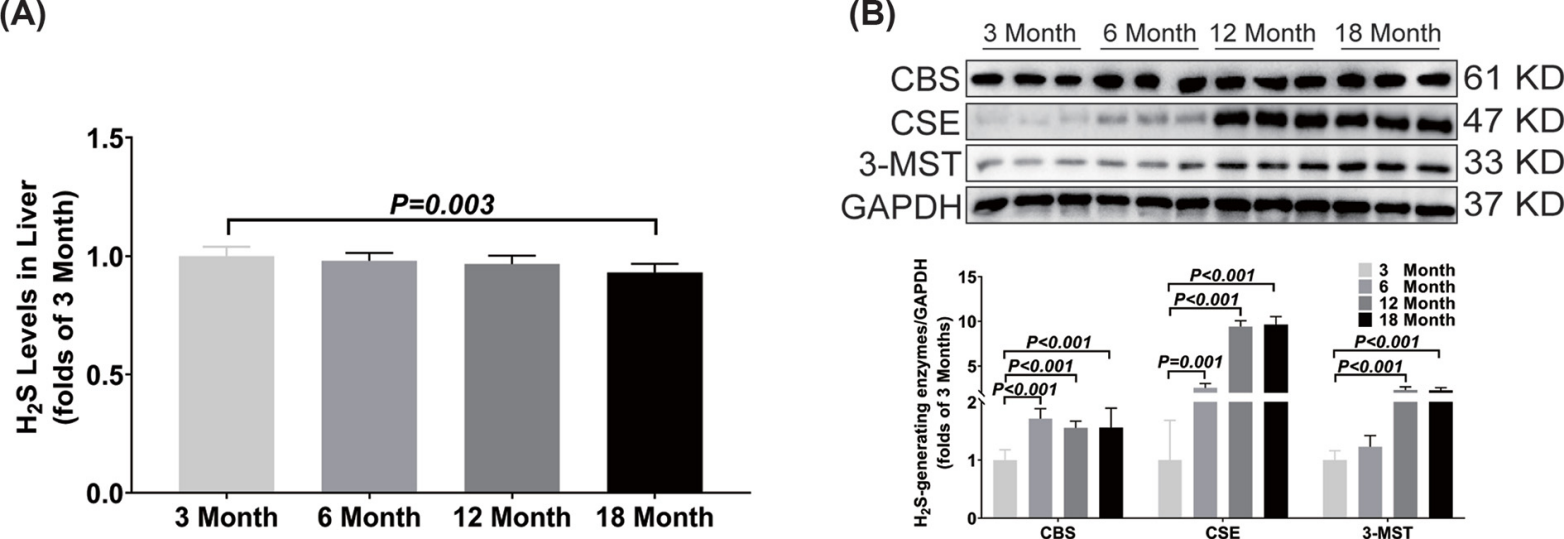
Changes of H_2_S content and H_2_S-generating enzymes expression in liver during aging (**A**) LC-MS/MS was used to detect the content of H_2_S in liver. (**B**) Western blotting images and bar graph comparing the expression levels of CBS, CSE and 3-MST expression levels in liver. Results are means ± SD, *n*=6. A *P* of <0.05 was considered significant.

In liver, the H_2_S concentration was only decreased in the 18-month-old group as compared with the 3-month-old group (Figure 2A). The CSE and CBS expressions were significantly up-regulated from 6 months old and the 3-MST expression was significantly up-regulated from 12 months old (Figure 2B).

**Figure 3:**
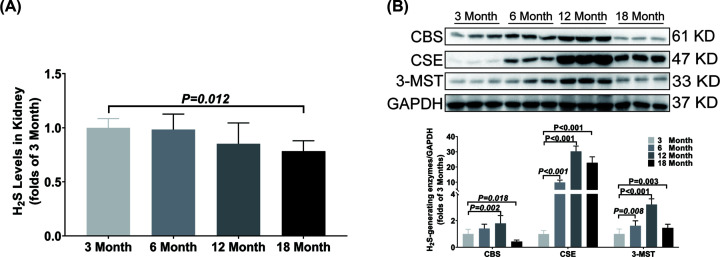
Changes of H_2_S content and H_2_S-generating enzymes expression in kidney during aging (**A**) LC-MS/MS was used to detect the content of H_2_S in kidney. (**B**) Western blotting images and bar graph comparing the expression levels of CBS, CSE and 3-MST expression levels in kidney. Results are means ± SD,* n*=6. A *P* of <0.05 was considered significant.

In kidney, the H_2_S concentration was only decreased in the 18-month-old group as compared with the 3-month-old group (Figure 3A). The CSE and 3-MST expressions were significantly up-regulated from 6 months old. Compared with the 3-month-old group, the CBS expression was significantly up-regulated in 12-month-old group but down-regulated in 18-month-old group (Figure 3B).

**Figure 4:**
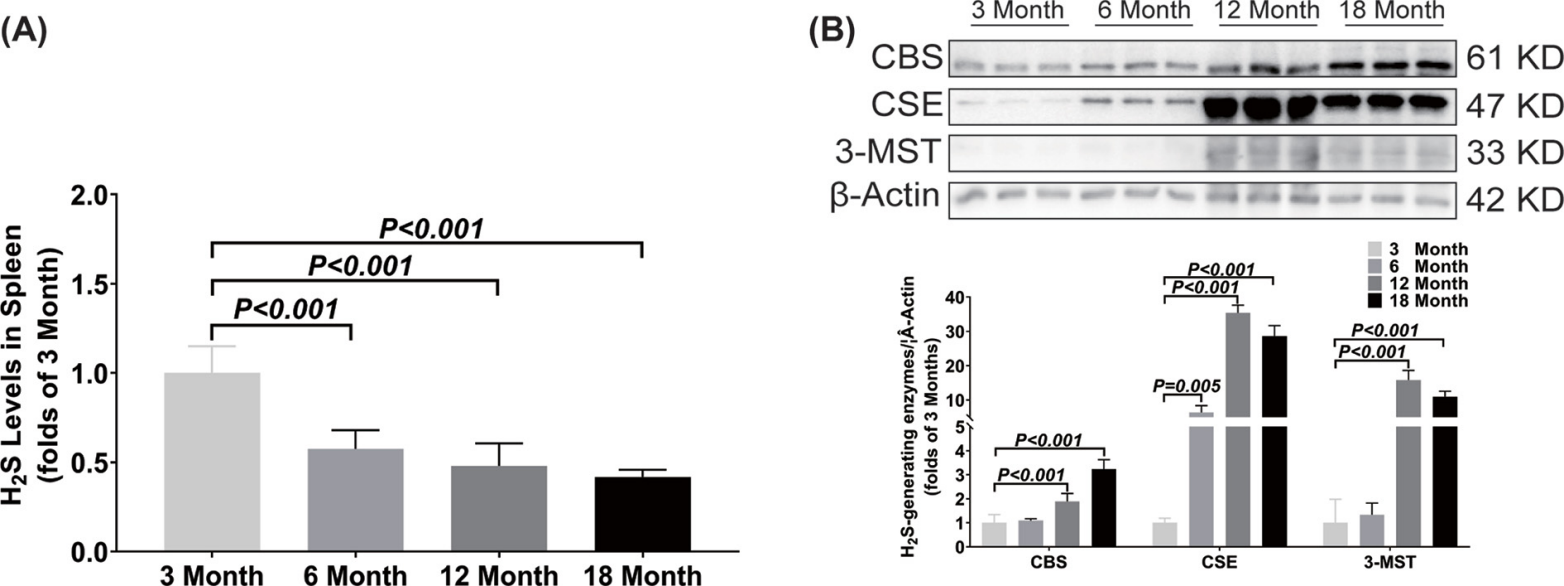
Changes of H_2_S content and H_2_S-generating enzymes expression in spleen during aging (**A**) LC-MS/MS was used to detect the content of H_2_S in spleen. (**B**) Western blotting images and bar graph comparing the expression levels of CBS, CSE and 3-MST expression levels in spleen. Results are means ± SD, *n*=6. A *P* of <0.05 was considered significant.

In spleen, the H_2_S concentration was significantly decreased from 6 months old (Figure 4A). The CSE expression was significantly up-regulated from 6 months old and the CBS and 3-MST expressions were significantly up-regulated from 12 months old (Figure 4B).

**Figure 5:**
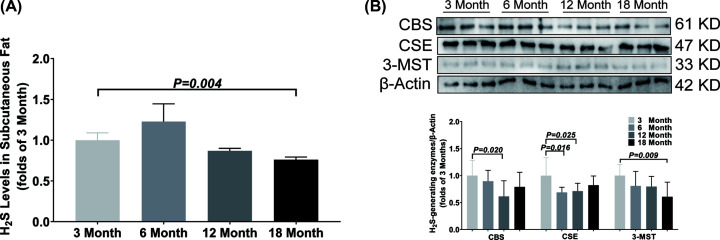
Changes of H_2_S content and H_2_S-generating enzymes expression in subcutaneous fat during aging (**A**) LC-MS/MS was used to detect the content of H_2_S in subcutaneous fat. (**B**) Western blotting images and bar graph comparing the expression levels of CBS, CSE and 3-MST expression levels in subcutaneous fat. Results are means ± SD, *n*=6. A *P* of <0.05 was considered significant.

In subcutaneous fat, the H2S concentration was only decreased in the 18-month-old group as compared with the 3-month-old group (Figure 5A). Compared with the 3-month-old group, the CBS expression was only down-regulated in 12-month-old group, the 3-MST expression was only down-regulated in 18-month-old group and the CSE was down-regulated both in 6-month-old and 12-month-old groups (Figure 5B).

**Figure 6:**
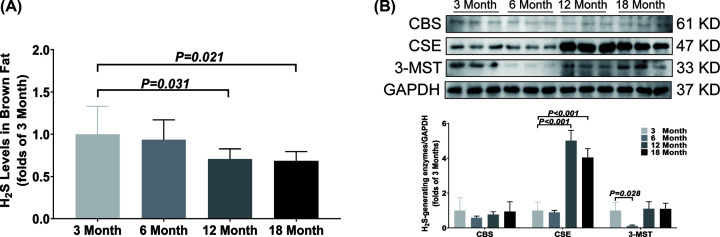
Changes of H_2_S content and H_2_S-generating enzymes expression in brown fat during aging (**A**) LC-MS/MS was used to detect the content of H_2_S in brown fat. (**B**) Western blotting images and bar graph comparing the expression levels of CBS, CSE and 3-MST expression levels in brown fat. Results are means ± SD, *n*=6. A *P* of <0.05 was considered significant.

In brown fat, the H_2_S concentration was significantly decreased from 12 months old (Figure 6A), while the CSE expression was significantly up-regulated at the meantime (Figure 6B). There was no significant difference in the expressions of CBS and 3-MST among the four different age groups except for the significant decrease of 3-MST in the 6-month-old group (Figure 6B).

**Figure 7:**
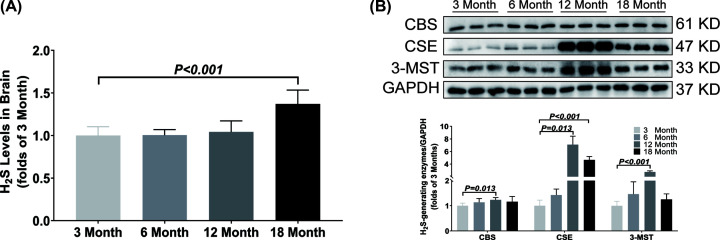
Changes of H_2_S content and H_2_S-generating enzymes expression in brain during aging (**A**) LC-MS/MS was used to detect the content of H_2_S in brain. (**B**) Western blotting images and bar graph comparing the expression levels of CBS, CSE and 3-MST expression levels in brain. Results are means ± SD, *n*=6. A *P* of <0.05 was considered significant.

In brain, the H_2_S concentration was significantly increased in the 18-month-old group as compared with the 3-month-old group (Figure 7A). The CSE expression was significantly up-regulated from 12 months old and the CBS and 3-MST expressions were only up-regulated in 12-month-old group (Figure 7B).

**Figure 8:**
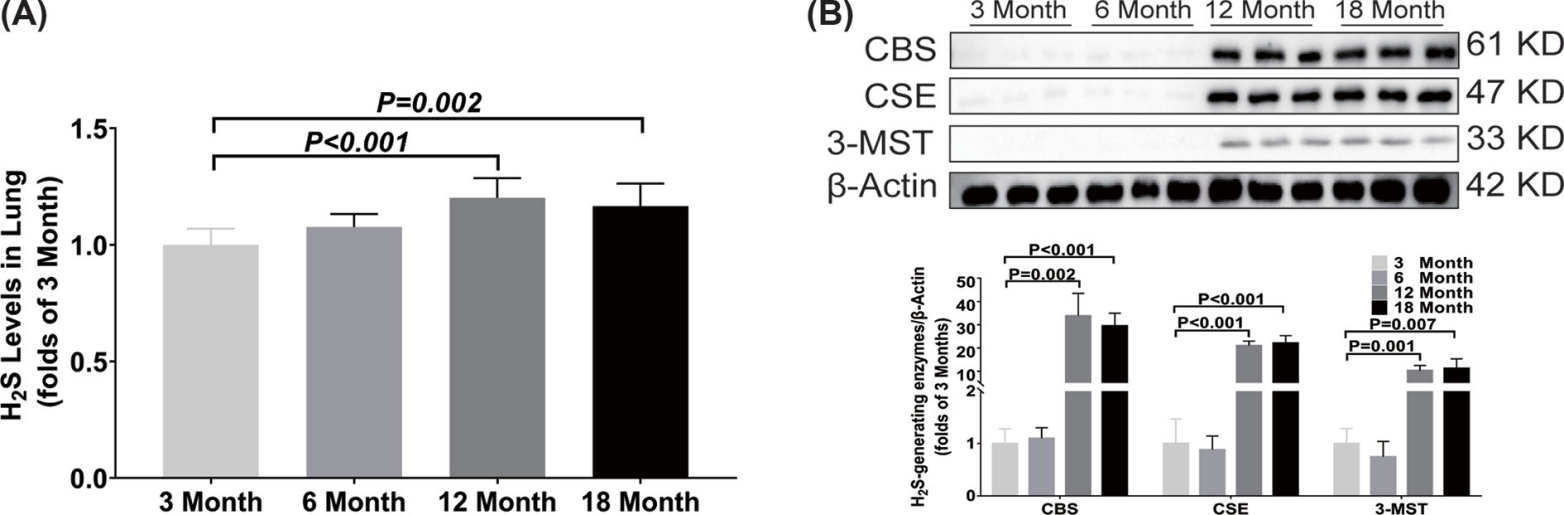
Changes of H_2_S content and H_2_S-generating enzymes expression in lung during aging (**A**) LC-MS/MS was used to detect the content of H_2_S in lung. (**B**) Western blotting images and bar graph comparing the expression levels of CBS, CSE and 3-MST expression levels in lung. Results are means ± SD, *n*=6. A *P* of <0.05 was considered significant.

In lung, the H_2_S concentration was significantly increased from 12 months old (Figure 8A), and the CBS and CSE 3-MST expressions were also significantly up-regulated from 12 months old (Figure 8B).

**Figure 9:**
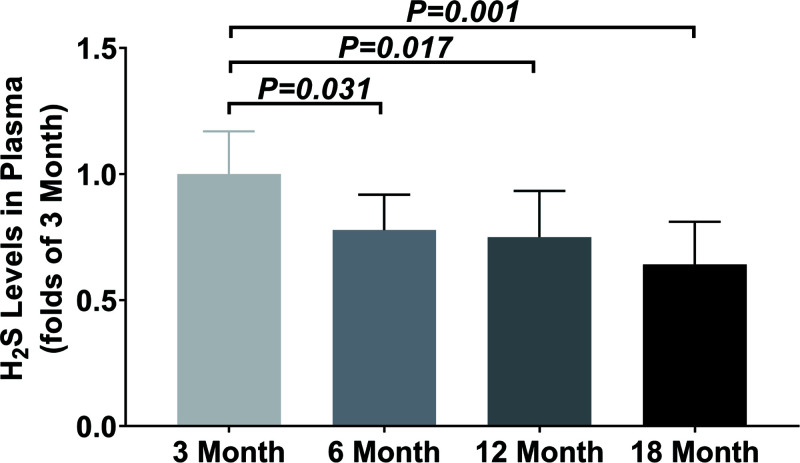
Changes of H_2_S content in plasma during LC-MS/MS was used to detect the content of H_2_S in plasma. Results are means ± SD, *n*=6. A *P* of <0.05 was considered significant.

In addition, the concentrations of H_2_S in the plasma were significantly decreased from 6 months old (Figure 9).

In aorta, there was no significant difference in the concentrations of H_2_S (Supplementary Figure S1A) and the expressions of 3-MST among the four different age groups. The CSE were significantly up-regulated from 6 months old and the CBS expression was only up-regulated in 12-month-old group as compared with the 3-month-old group (Supplementary Figure S1B).

In skeletal muscle, there was no significant difference in the concentrations of H_2_S (Supplementary Figure S2A), the expressions of CBS (Supplementary Figure S2B) among the four different age groups. The CSE expression was significantly up-regulated from 6 months old and the 3-MST expression was up-regulated in 6-month-old and 18-month-old group as compared with the 3-month-old group (Supplementary Figure S2B).

In colon, there was no significant difference in the concentrations of H_2_S among the four different age groups (Supplementary Figure S3A). The CSE expression was significantly up-regulated from 6 months old and the CBS and 3-MST expression was only up-regulated in 6-month-old group as compared with the 3-month-old group (Supplementary Figure S3B).

In skin, the H_2_S concentration was not significantly different among the four different age groups (Supplementary Figure S4A). Compared with the 3-month-old group, the CBS expression was significantly down-regulated in 12-month-old group but up-regulated in 18-month-old group. The CSE expression was significantly up-regulated from 6 months old and the 3-MST expression was only up-regulated in 18-month-old group (Supplementary Figure S4B).

In cerebellum, there was no significant difference in the concentrations of H_2_S among the four different age groups, although the mean value of its concentration in 18-month-old group was increased (Supplementary Figure S5A). The CBS expression was not significantly different, while the CSE and 3-MST expressions were significantly up-regulated from 12 months old (Supplementary Figure S5B).

## Discussion

In the present study, we investigated four cross-sectional age groups (3, 6, 12 and 18 months of age) spanning the lifespan of male C57BL/6J mice to systematically analyze the H_2_S concentration in plasma and 13 tissues; meanwhile, the expressions of three H_2_S-generating enzymes (CSE, CBS and 3-MST) from the 13 tissues also were detected. We found that (1) the H_2_S concentrations were decreased asynchronously with the aging process in plasma, heart, liver, kidney, spleen, subcutaneous fat and brown fat; were increased in brain and lung; and remained unchanged in aorta, skeletal muscle, colon, skin and cerebellum. (2) In most tissues, at least one of the three H_2_S-generating enzymes expressions were compensatively up-regulated with the aging process except in the subcutaneous fat.

Previous studies have shown that chronic nephritis and sepsis can lead to a decrease in the ability of rat kidneys to catalyze the production of hydrogen sulfide, resulting in a decrease in hydrogen sulfide levels in the kidneys and impaired kidney function. Supplementing with exogenous hydrogen sulfide can improve kidney function disorders caused by the disease [18–20]. Various cardiovascular diseases can also lead to a decrease in hydrogen sulfide levels in the cardiovascular system, such as heart failure and myocardial ischemia–reperfusion. Supplementing with exogenous hydrogen sulfide can also improve cardiovascular system diseases [21–23].

Aging is an inevitable and irreversible complex process that is associated with a progressive physiological function decline and physical deterioration, resulting in an increased risk of disease and death. The proposed mechanisms that contribute to the aging process and the development of age-related diseases include free radical accumulation, proteostasis loss, DNA damage, and chronic inflammation [24–27]. As a newly discovered endogenous gasotransmitter, exogenous administration of H_2_S donors or promotion of endogenous H_2_S production has been reported to provide multiple beneficial effects on the regulation of aging including extended longevity in different species by scavenging free radical, restoring proteostasis, attenuating DNA damage and inhibiting inflammation [28–31]. Sustaining evidence showed circulating H_2_S levels were significant age-associated reductions in both males and females [12] and persulfidation, a novel post-translational modification induced by H_2_S, was diminished during aging [32]. In consistent with the above research, we measured plasma H_2_S levels from male C57BL/6J mice (*n*=6, aged 3, 6, 12 and 18 months) and found that the plasma H_2_S levels were significantly decreased from 6 months old.

Detection methods for H_2_S, including methylene blue spectroscopy, gas chromatography and monobromodiphenyl (MMB) method [33]. After synthesized in mammalian tissues, free H_2_S rapidly traveled through cell membranes by simple diffusion and was dissolved in extracellular fluid and plasma about 81.5% existing as HS^−^ and 18.5% as H_2_S [34]. Often for convenience the sum of dissolved H_2_S and HS^−^ were referred to as ‘H_2_S’. Apart from the free state, H_2_S was also stored in forms of acid labile and bound sulfane sulfur pools in mammalian cells [35]. Acid-labile sulfur pool released H_2_S under acidic conditions and bound sulfane sulfur pool released H_2_S under reducing conditions. The methylene blue method was one of the commonly used spectrometric methods for measuring circulating H_2_S [36]. However, in this method, strong acid was used which led to overestimation of H_2_S content due to the release of H_2_S from acid-labile sulfur in addition to free H_2_S.

Given tissues aged at different rates and differences in H_2_S concentrations among tissues, we investigated the H_2_S concentration in thirteen tissues from four age groups (3, 6, 12 and 18 months of age) and found that the H_2_S concentration did not change in aorta, skeletal muscle, colon, skin and cerebellum. However, there was an asynchronously age-dependent decrease in H_2_S concentration in heart, liver, kidney, spleen, subcutaneous fat and brown fat, while H_2_S concentrations were increased in brain and lung. A recent study found that the main organs which controlled metabolic function, including fat and liver cells, had a faster rate of aging, in contrast, neuronal cells in the brain had a more slowly rate of aging [37]. Correspondingly, our results showed that the H_2_S concentration was decreased in the tissues with faster aging rate mentioned above, and was increased in the tissues with slower aging rate, which indicated that tissues with high metabolic rates and rich blood flow were more prone to age-related declines in H_2_S concentration.

In mammals, H_2_S is generated endogenously through the action of three main enzymes: CSE, CBS and 3-MST. Although the tissue relative distribution of the three enzymes differs, they are apparent overlap and widespread in tissues and cells. In the present study, we found that three H_2_S-generating enzymes were widely expressed in the thirteen tissues and at least one of them expression was up-regulated with the aging process in most tissues, among which the up-regulation of CSE was the most obvious. Aging led to increased ROS production and H_2_S displayed antioxidant effects through direct quenching of ROS [38]. If H_2_S was not replenished, including endogenously and exogenously in time, this would lead to age-related declines in H_2_S concentrations and the increase of ROS levels. On the one hand, excessive ROS could inhibit the activity of the H_2_S-generating enzymes, especially in tissues with high metabolic rates, such as the heart. On the other hand, it could cause compensatory up-regulation of the H_2_S-generating enzymes, although this compensation did not actually increase H_2_S levels. In particular, as an inducible H_2_S generating enzyme, CSE expression was highly inducible by a range of stimuli including oxidative stress [39]. It was reported that NADPH promoted CSE transcription and enhanced its protein expression via a heme-regulated inhibitor kinase/eIF2α/activating transcription factor-4 signaling pathway [40]. In addition it was observed that nuclear factor (erythroid-derived 2)-like 2, a transcription factor that controlled the basal and inducible expression of an array of antioxidant enzymes, up-regulated both CBS and CSE expression in response to oxidative stress [41,42]. A chronic low-grade inflammation was another mainly hallmark of aging and was strongly associated with various age-related pathologies. Nuclear factor-κB, a transcription factor that was one of the most important molecules linking chronic inflammation to aging, was found to be a positive regulator of 3-MST mRNA expression [43]. LPS stimulation also led to increased association of SP1 with the CSE promoter which elevated H_2_S production in macrophages [44]. Our results were also in line with previous studies which showed a significant decrease in H_2_S concentrations but a higher CSE expression in tissues [45,46]. On the contrary, mouse embryonic fibroblasts isolated from CSE knockout mice displayed increased oxidative stress and accelerated cellular senescence, while incubation of the cells with sodium hydrosulfide (NaHS, a H2S donor) attenuated oxidative stress and protected against cellular senescence via S-sulfhydration of Keap1 and activation of Nrf2 [47]. In mice, exogenous supplementation of H_2_S precursors, GYY4137 and NaHS, reversed vascular aging by increasing SIRT1 activity to protect against oxidative stress [48]. In addition, dietary restriction also played its antiaging effects by promoting endogenous H_2_S production [49,50].

It has been clearly established that H_2_S can be non-enzymatically synthesized, Previous studies have shown that sulfur-containing amino acids can produce H_2_S under physiological conditions in the presence of synergistic catalysis of vitamin B6, pyridoxal (phosphate) and iron. However, enzymatic generation of hydrogen sulfide is the main pathway in general tissues [51,52].

There were some limitations in our study. Firstly, in present study, we only reported the phenomenon of asynchronous changes in H_2_S concentration during age, but did not further explore whether aging led to the change in H_2_S concentration or the change in H_2_S concentration led to aging. Secondly, due to the high reactivity and rapid tissue catabolism of H_2_S under biological environments, the MBB method we used could not real-timely determinate the exact concentration of H_2_S and its subcellular distribution. Thirdly, H_2_S was biosynthesized in mammals via both enzymatic and nonenzymatic pathways and was mainly metabolized by three mechanisms: oxidation, methylation and sulfhemoglobin formation by the binding to hemoglobin. In our study, we only observed the changes of three H_2_S-generating enzymes during aging, but did not investigate the non-enzymatic pathways and metabolic pathways, which need to be further explored.

In conclusion, we found that the H_2_S concentrations were decreased asynchronously with the aging process in plasma, heart, liver, kidney, spleen, subcutaneous fat and brown fat, and were increased in brain and lung. At least one of the three H_2_S-generating enzymes expression was compensatively up-regulated with the aging process in most tissues, among which the up-regulation of CSE was the most obvious.

## Supplementary Material

Supplementary Figures S1-S5

## Data Availability

All data supported the findings of this study can be available from the corresponding author upon reasonable request.

## Abbreviations

3-MST: 3-mercaptopyruvate sulfurtransferase
CBS: cystathionine β-synthase
CSE: cystathionine γ-lyase
MMB: monobromodiphenyl
ROS: reactive oxygen species
