# Supplementary material for: Asynchronous changes of hydrogen sulfide and its generating enzymes in most tissues with the aging process
Source: Biosci Rep. 2024 Oct 11;44(10):BSR20240320. doi: 10.1042/BSR20240320 (PMC11473966; doi:10.1042/BSR20240320)
Supplement: Supplementary Figures S1-S5 [file BSR-2024-0320_supp.zip › BSR-2024-0320_supp.pdf]

Supplementary Figure legends

Supplementary Fig.1 Changes of H_2S content and H_2S-generating enzymes expression in artery during aging. (A) LC-MS/MS was used to detect the content of H_2S in artery. (B) Western blotting images and bar graph comparing the expression levels of CBS, CSE and 3-MST expression levels in artery. Results are means ± SD, n=6. A P of <0.05 was considered significant.

Supplementary Fig.2 Changes of H_2S content and H_2S-generating enzymes expression in skeletal muscle during aging. (A) LC-MS/MS was used to detect the content of H_2S in skeletal muscle. (B) Western blotting images and bar graph comparing the expression levels of CBS, CSE and 3-MST expression levels in skeletal muscle. Results are means ± SD, n=6. A *P* of <0.05 was considered significant.

Supplementary Fig.3 Changes of H_2S content and H_2S-generating enzymes expression in colon during aging. (A) LC-MS/MS was used to detect the content of H_2S in colon. (B) Western blotting images and bar graph comparing the expression levels of CBS, CSE and 3-MST expression levels in colon. Results are means ± SD, n=6. A *P* of <0.05 was considered significant.

Supplementary Fig.4 Changes of H_2S content and H_2S-generating enzymes expression in skin during aging. (A) LC-MS/MS was used to detect the content of H_2S in skin. (B) Western blotting images and bar graph comparing the expression levels of CBS, CSE and 3-MST expression levels in skin. Results are means ± SD, n=6. A *P* of <0.05 was considered significant.

Supplementary Fig.5 Changes of H_2S content and H_2S-generating enzymes expression in cerebellum during aging. (A) LC-MS/MS was used to detect the content of H_2S in cerebellum. (B) Western blotting images and bar graph comparing the expression levels of CBS, CSE and 3-MST expression levels in cerebellum. Results are means ± SD, n=6. A *P* of <0.05 was considered significant.

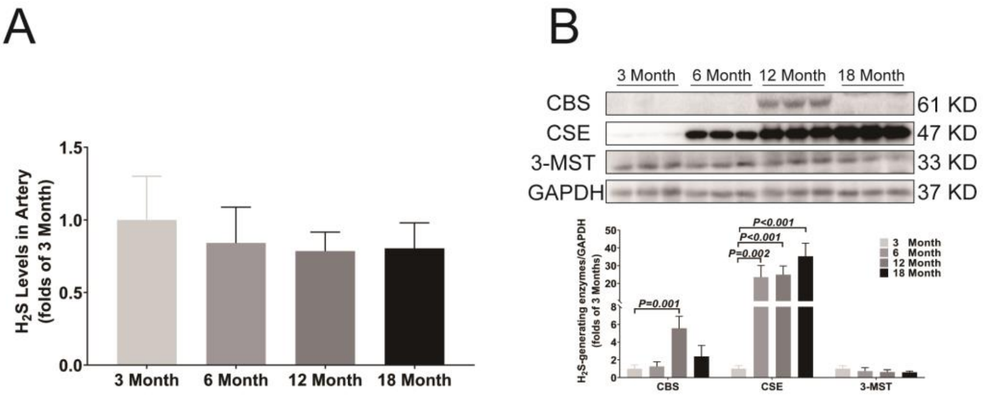

Supplementary Fig.1 Changes of H_2S content and H_2S-generating enzymes expression in artery during aging. (A) LC-MS/MS was used to detect the content of H_2S in artery. (B) Western blotting images and bar graph comparing the expression levels of CBS, CSE and 3-MST expression levels in artery.

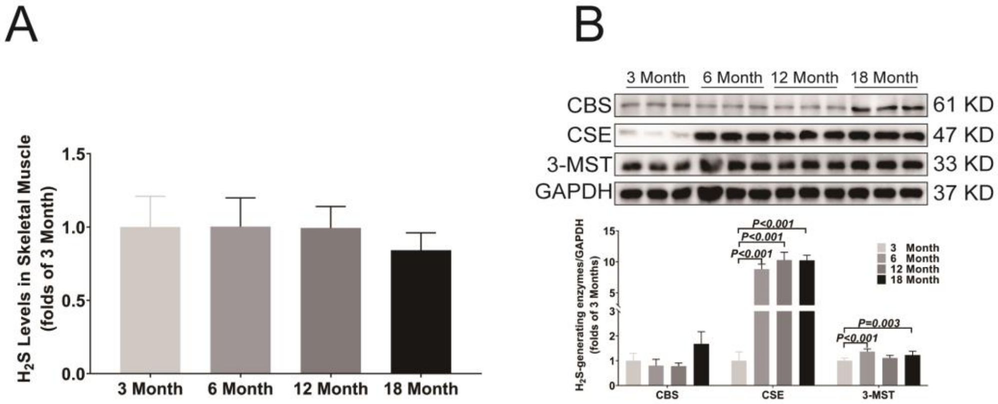

Supplementary Fig.2 Changes of H_2S content and H_2S-generating enzymes expression in skeletal muscle during aging. (A) LC-MS/MS was used to detect the content of H_2S in skeletal muscle. (B) Western blotting images and bar graph comparing the expression levels of CBS, CSE and 3-MST expression levels in skeletal muscle.

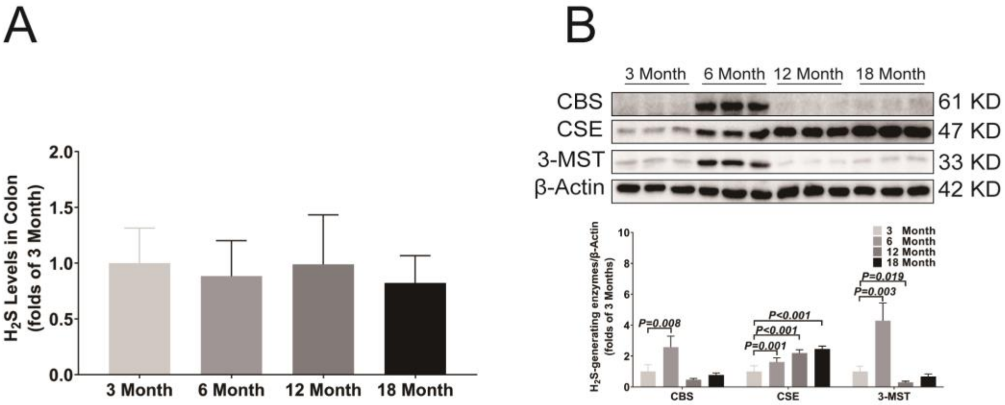

Supplementary Fig.3 Changes of H_2S content and H_2S-generating enzymes expression in colon during aging. (A) LC-MS/MS was used to detect the content of H_2S in colon. (B) Western blotting images and bar graph comparing the expression levels of CBS, CSE and 3-MST expression levels in colon.

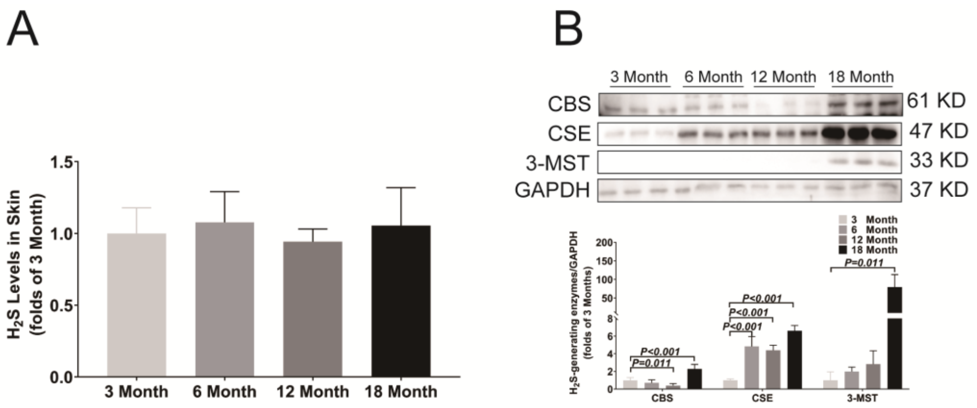

Supplementary Fig.4 Changes of H_2S content and H_2S-generating enzymes expression in skin during aging. (A) LC-MS/MS was used to detect the content of H_2S in skin. (B) Western blotting images and bar graph comparing the expression levels of CBS, CSE and 3-MST expression levels in skin.

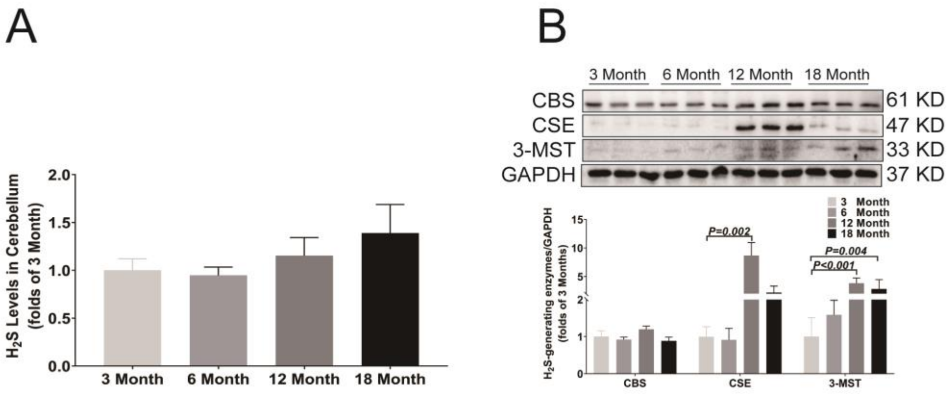

Supplementary Fig.5 Changes of H_2S content and H_2S-generating enzymes expression in cerebellum during aging. (A) LC-MS/MS was used to detect the content of H_2S in cerebellum. (B) Western blotting images and bar graph comparing the expression levels of CBS, CSE and 3-MST expression levels in cerebellum.
