# Supplementary figures and images for: Asynchronous changes of hydrogen sulfide and its generating enzymes in most tissues with the aging process
Source: Biosci Rep. 2024 Oct 11;44(10):BSR20240320. doi: 10.1042/BSR20240320 (PMC11473966; doi:10.1042/BSR20240320)

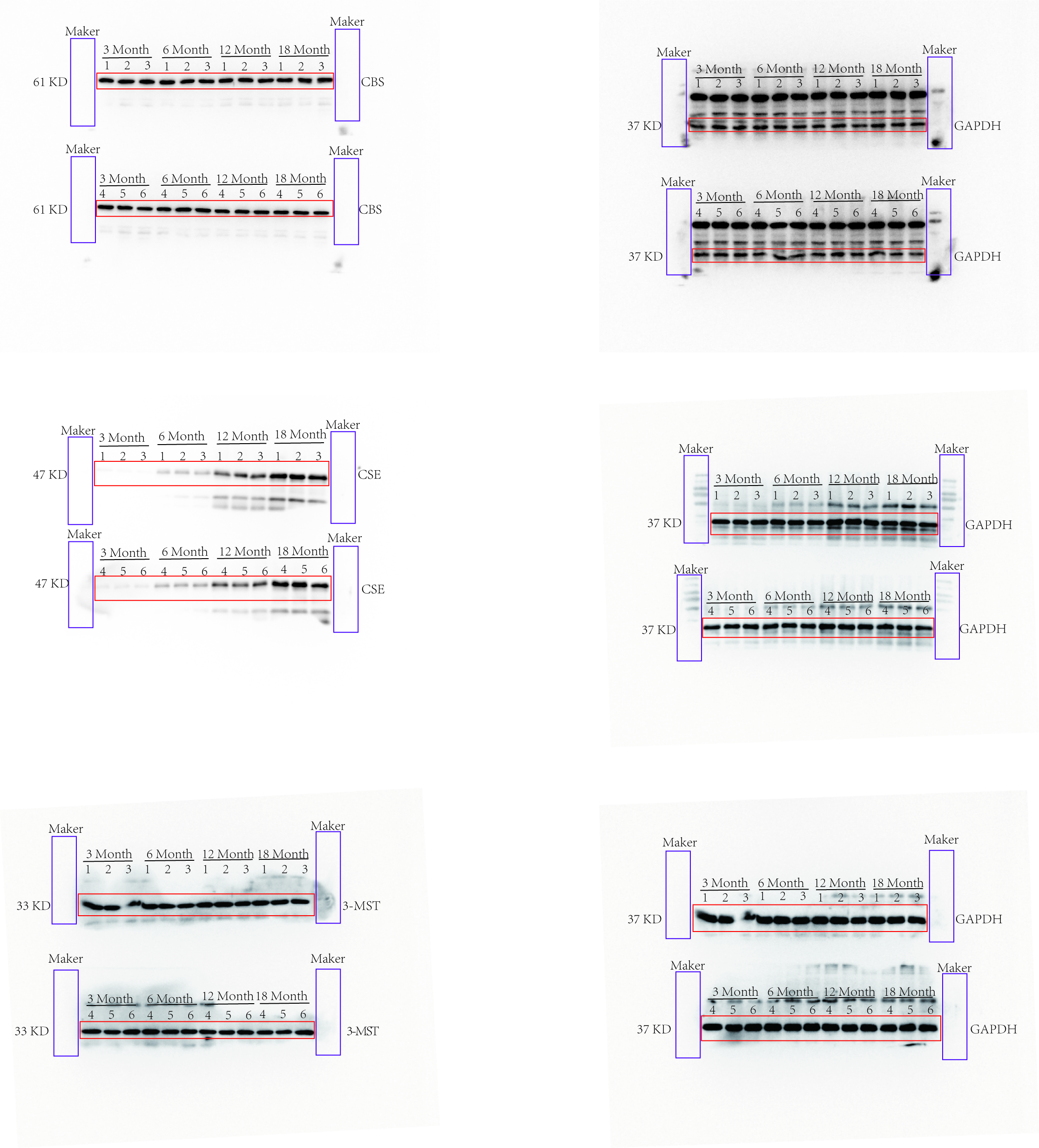

Supplement: Supplementary Figures S1-S5 [file BSR-2024-0320_supp.zip › BSR-2024-0320_supp1B.tif]

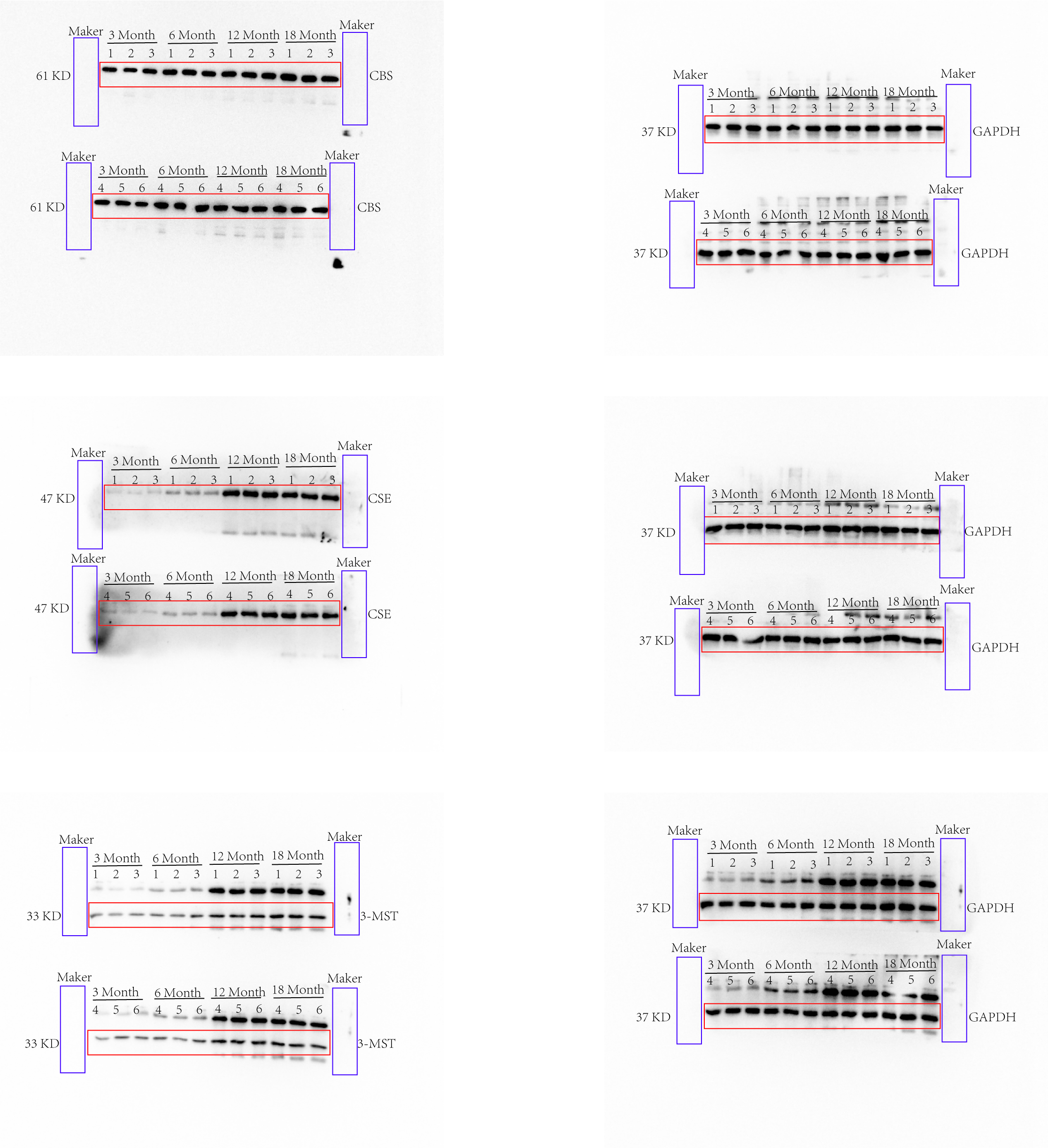

Supplement: Supplementary Figures S1-S5 [file BSR-2024-0320_supp.zip › BSR-2024-0320_supp2B.tif]

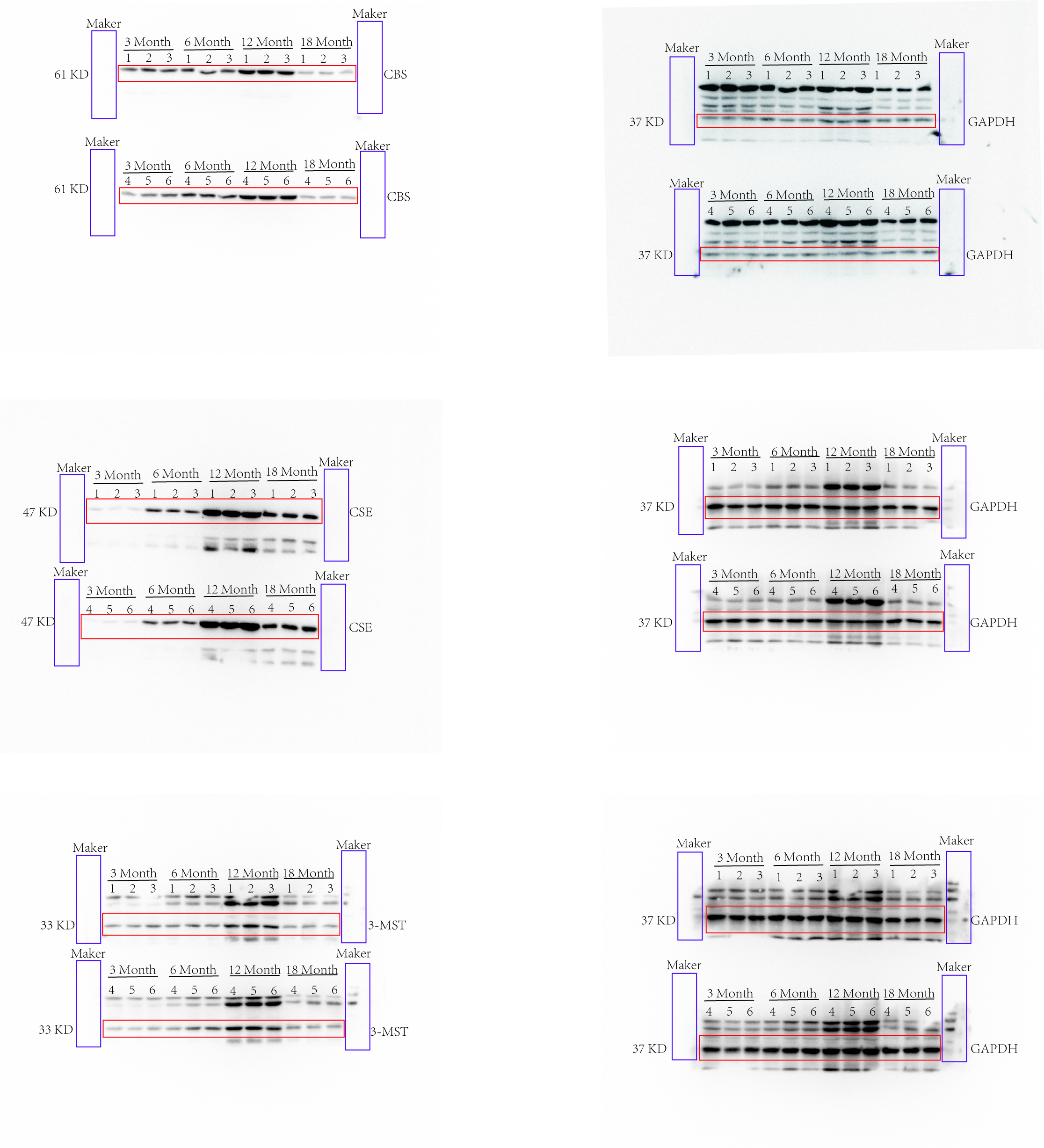

Supplement: Supplementary Figures S1-S5 [file BSR-2024-0320_supp.zip › BSR-2024-0320_supp3B.tif]

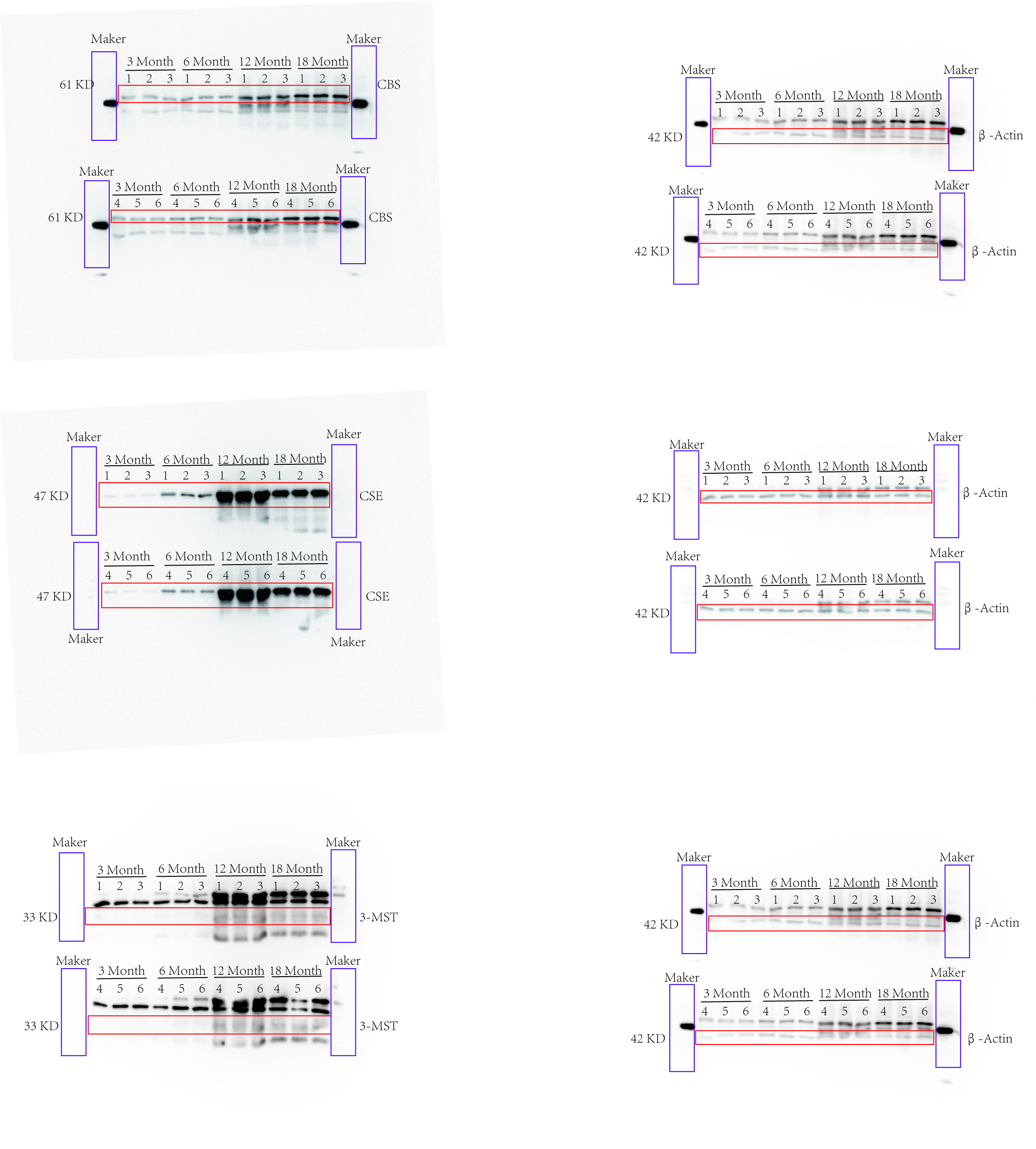

Supplement: Supplementary Figures S1-S5 [file BSR-2024-0320_supp.zip › BSR-2024-0320_supp4B.tif]

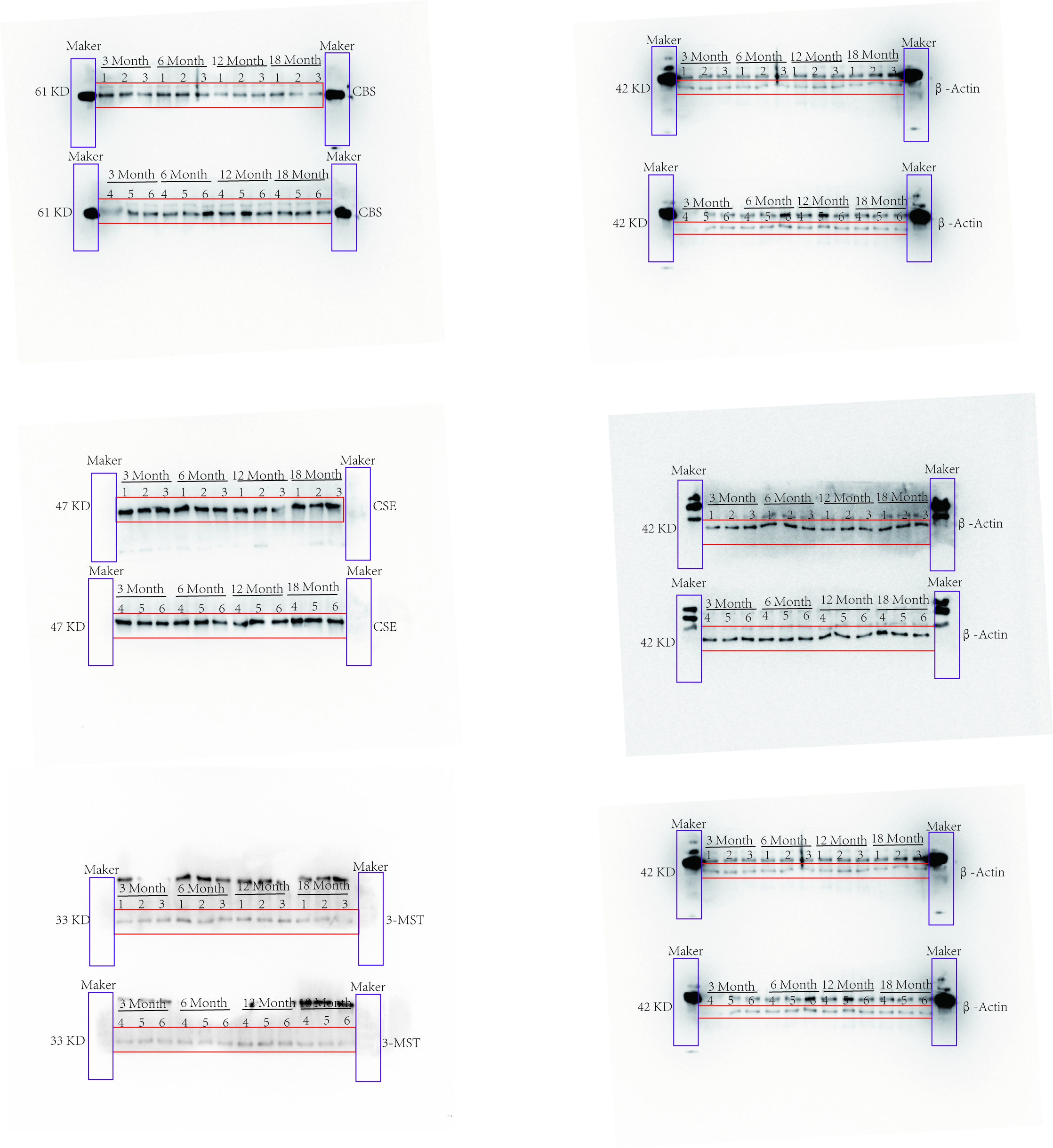

Supplement: Supplementary Figures S1-S5 [file BSR-2024-0320_supp.zip › BSR-2024-0320_supp5B.tif]

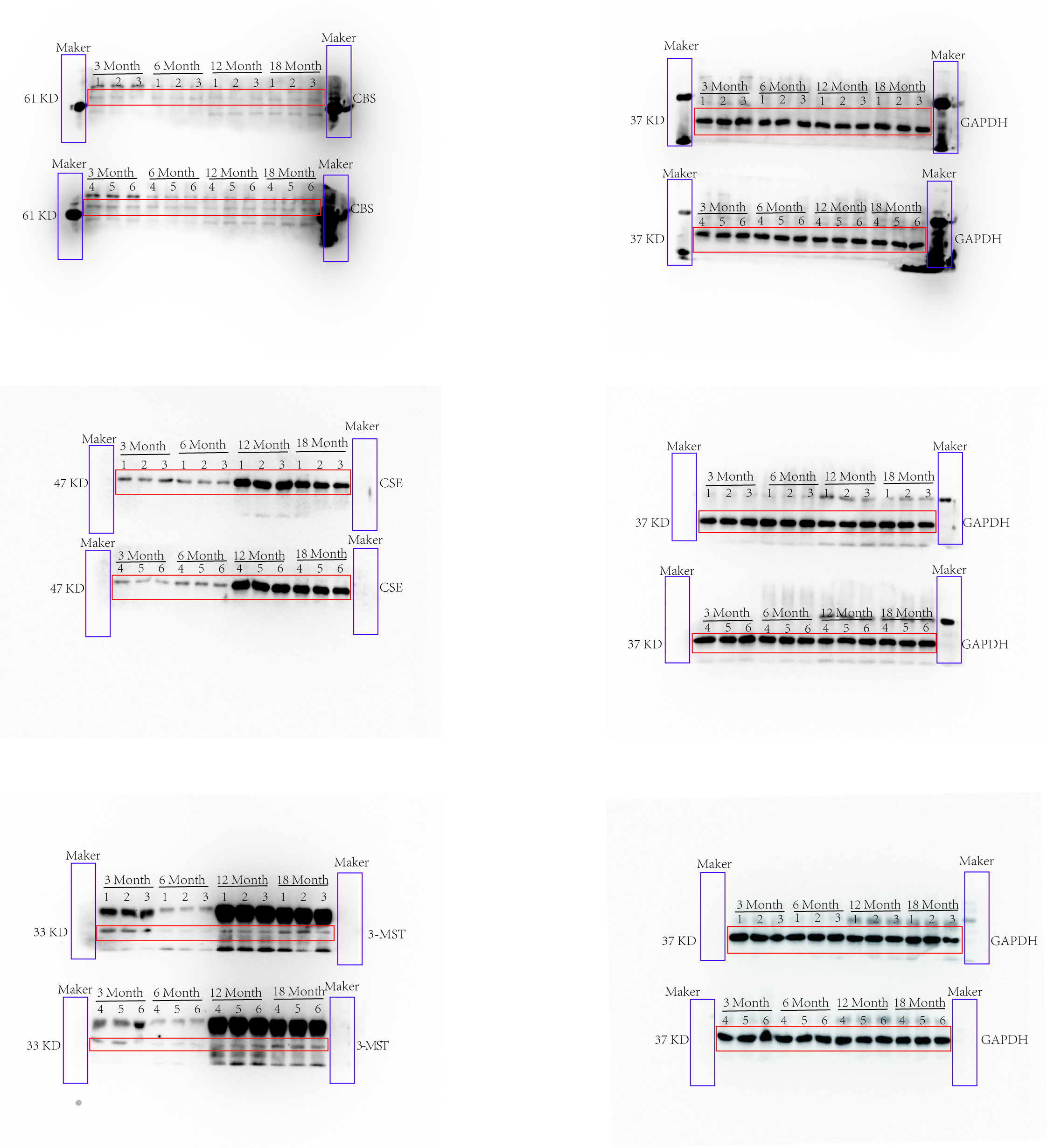

Supplement: Supplementary Figures S1-S5 [file BSR-2024-0320_supp.zip › BSR-2024-0320_supp6B.tif]

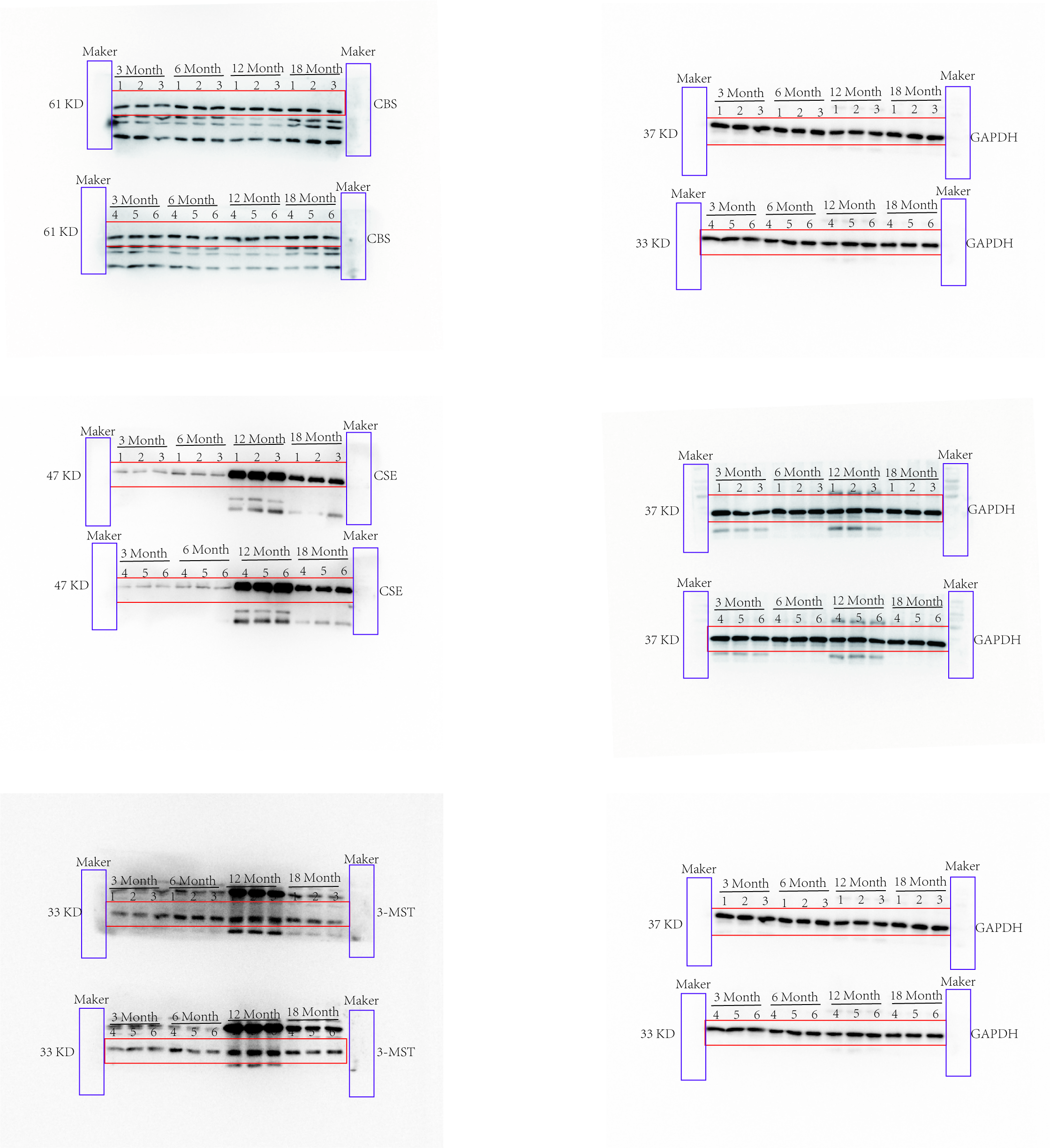

Supplement: Supplementary Figures S1-S5 [file BSR-2024-0320_supp.zip › BSR-2024-0320_supp7B.tif]

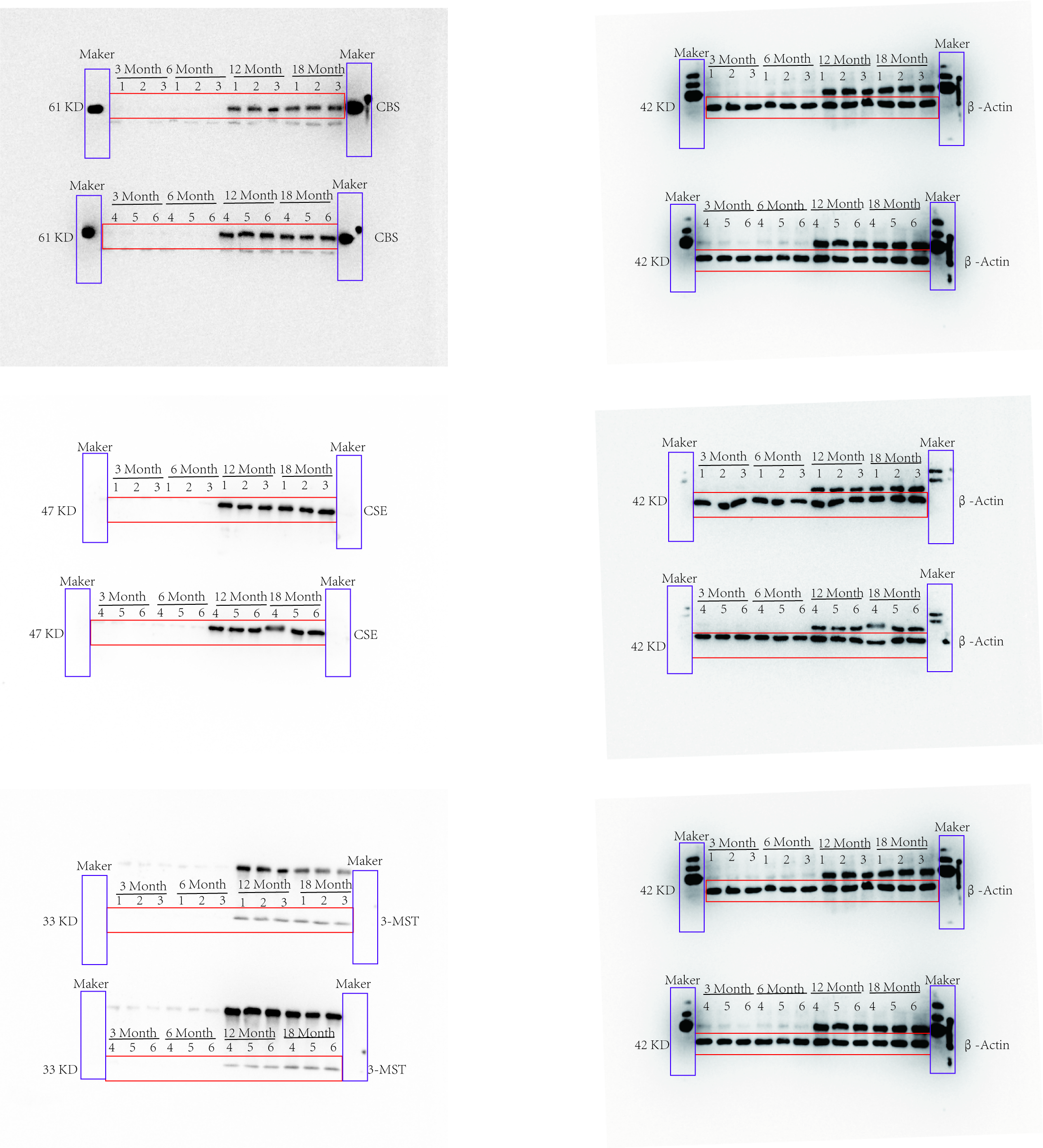

Supplement: Supplementary Figures S1-S5 [file BSR-2024-0320_supp.zip › BSR-2024-0320_supp8B.tif]

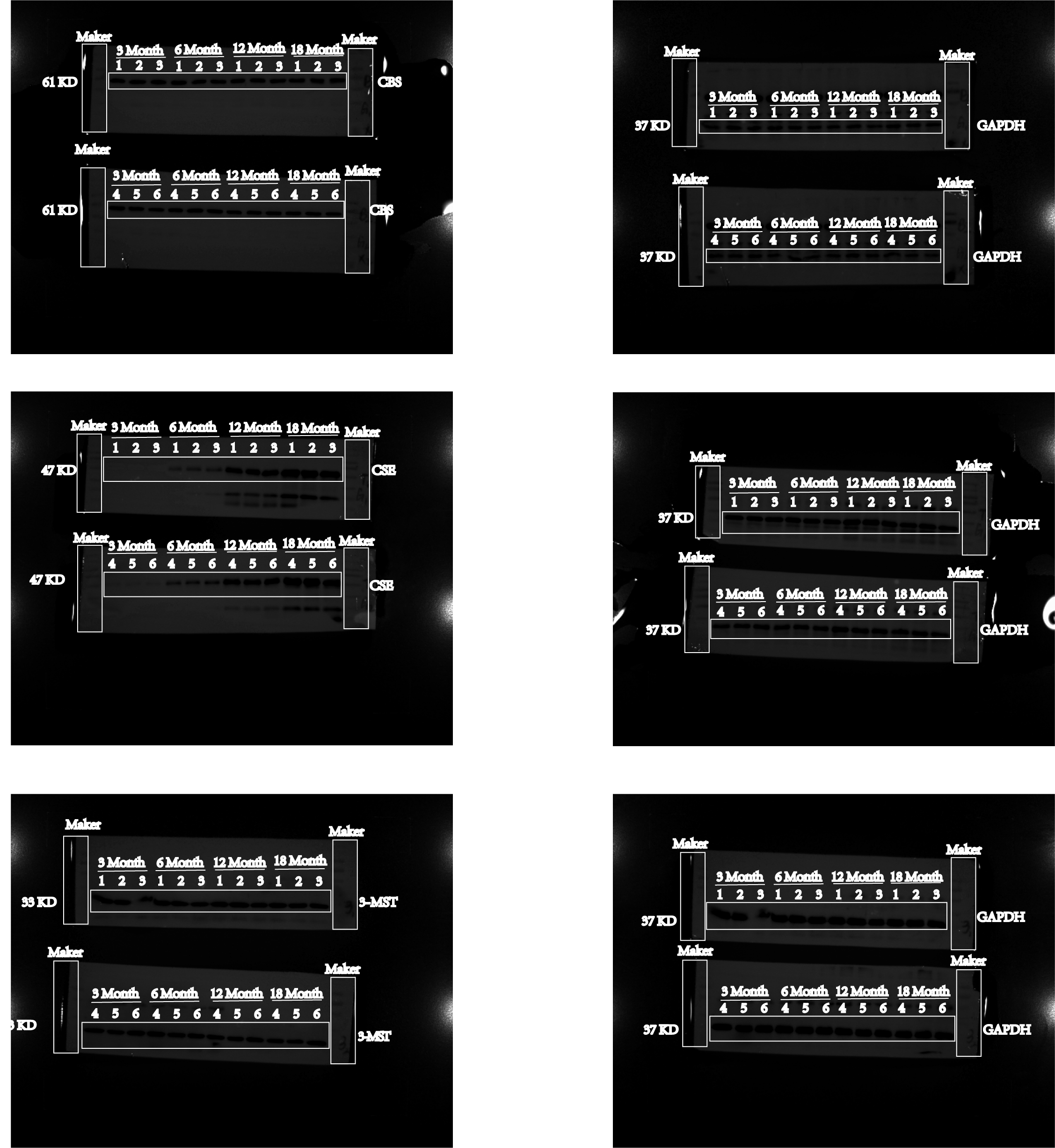

Supplement: Supplementary Figures S1-S5 [file BSR-2024-0320_supp.zip › BSR-2024-0320_suppo1B.tif]

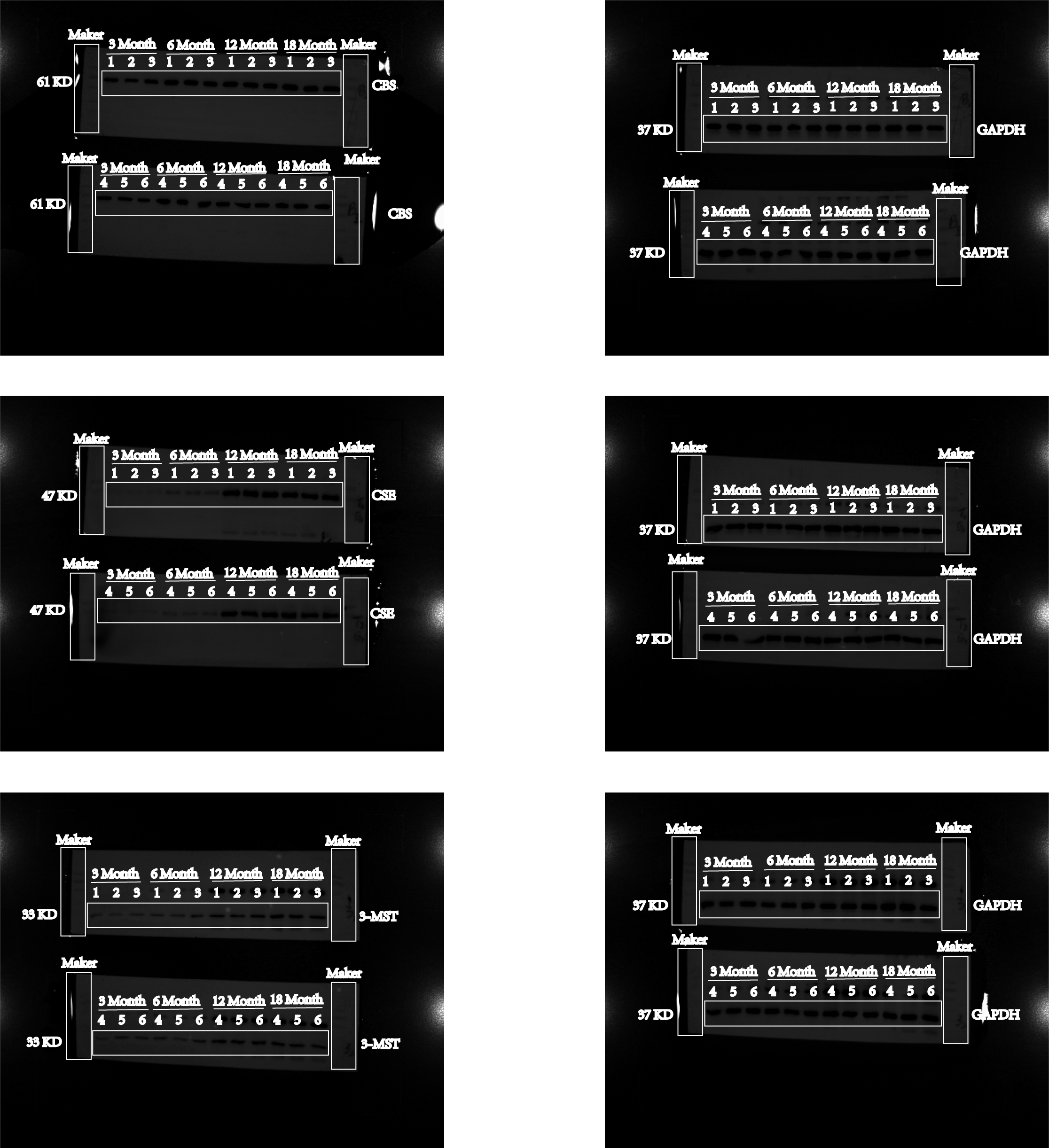

Supplement: Supplementary Figures S1-S5 [file BSR-2024-0320_supp.zip › BSR-2024-0320_suppo2B.tif]

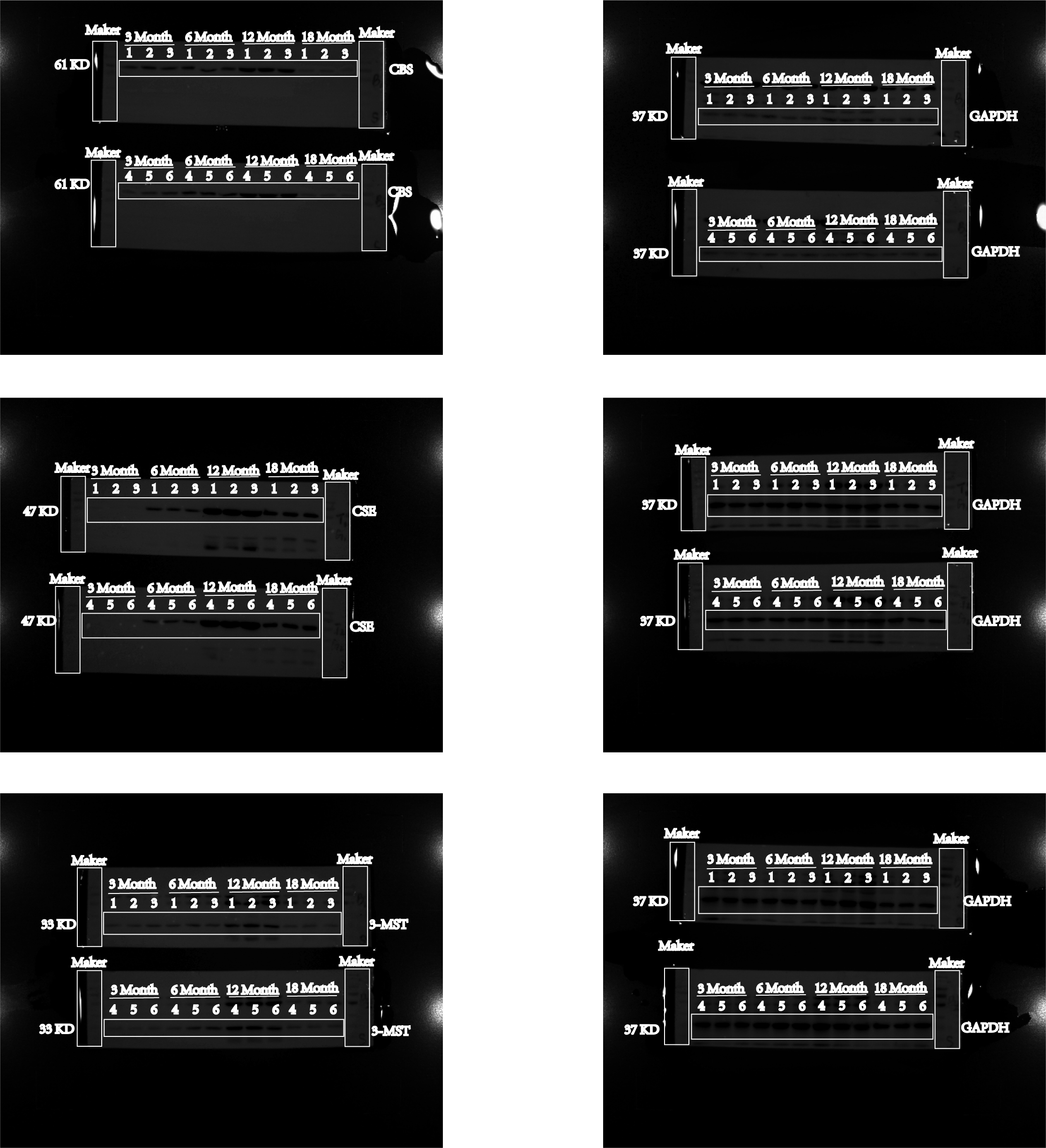

Supplement: Supplementary Figures S1-S5 [file BSR-2024-0320_supp.zip › BSR-2024-0320_suppo3B.tif]

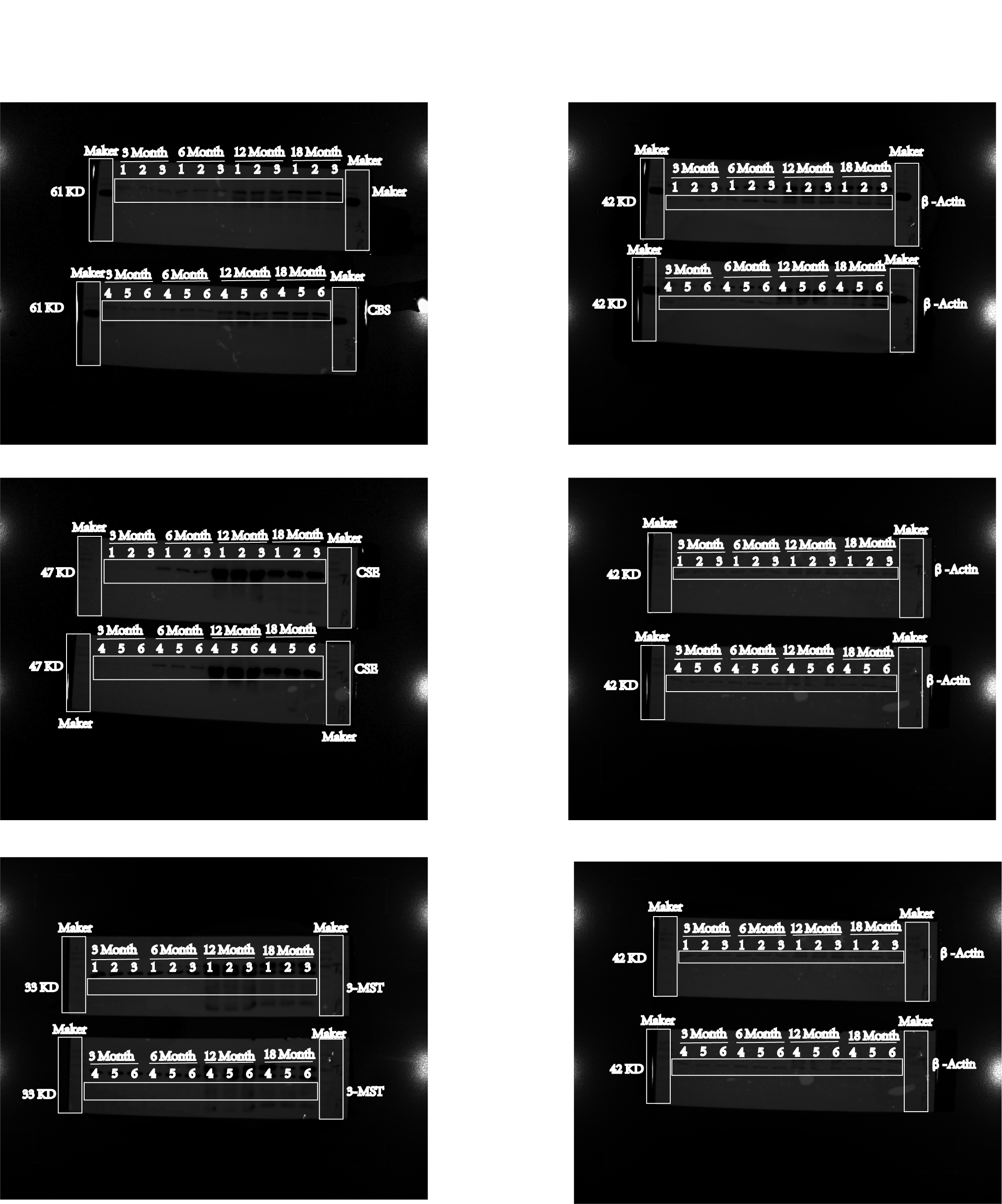

Supplement: Supplementary Figures S1-S5 [file BSR-2024-0320_supp.zip › BSR-2024-0320_suppo4B.tif]

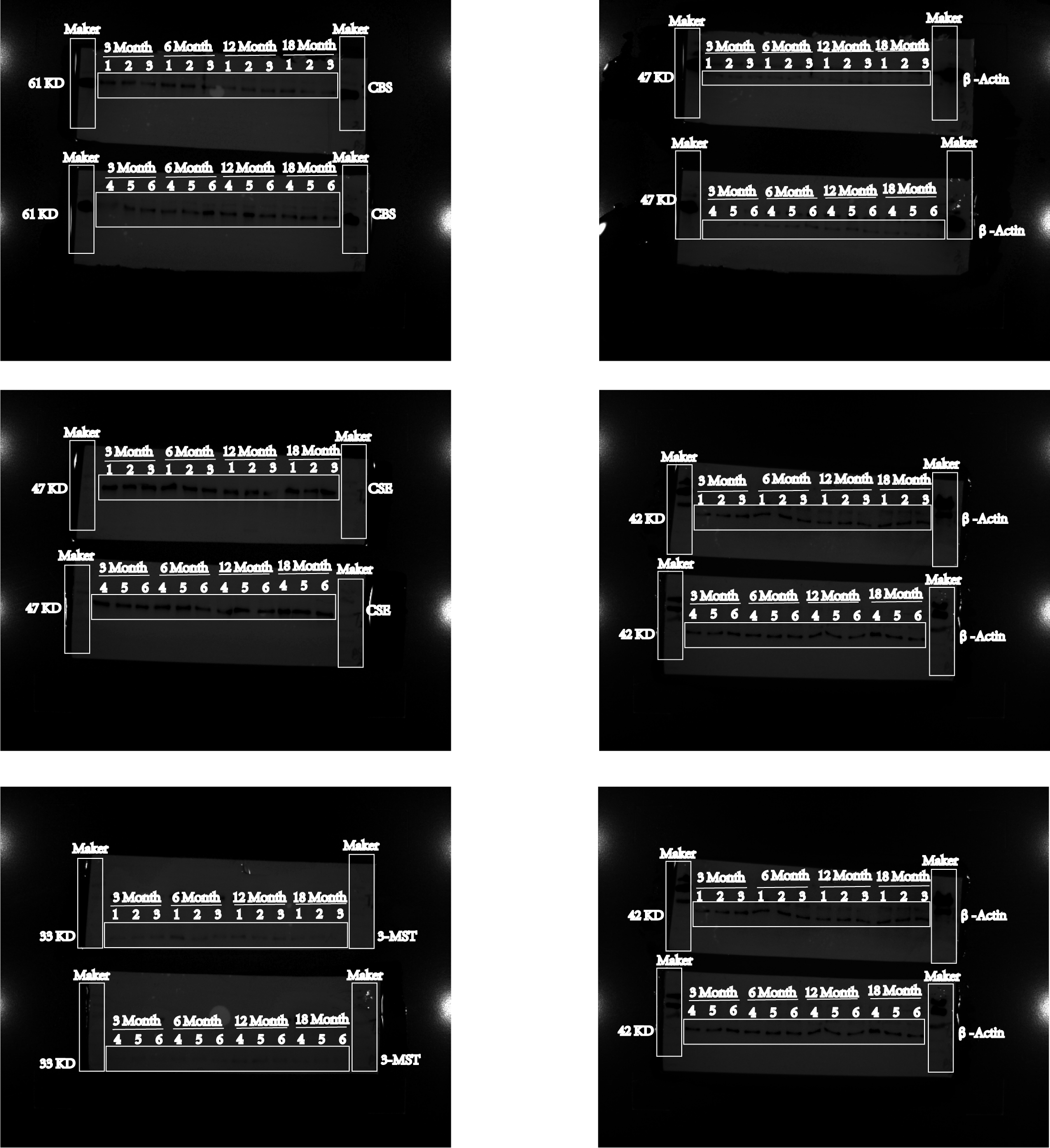

Supplement: Supplementary Figures S1-S5 [file BSR-2024-0320_supp.zip › BSR-2024-0320_suppo5B.tif]

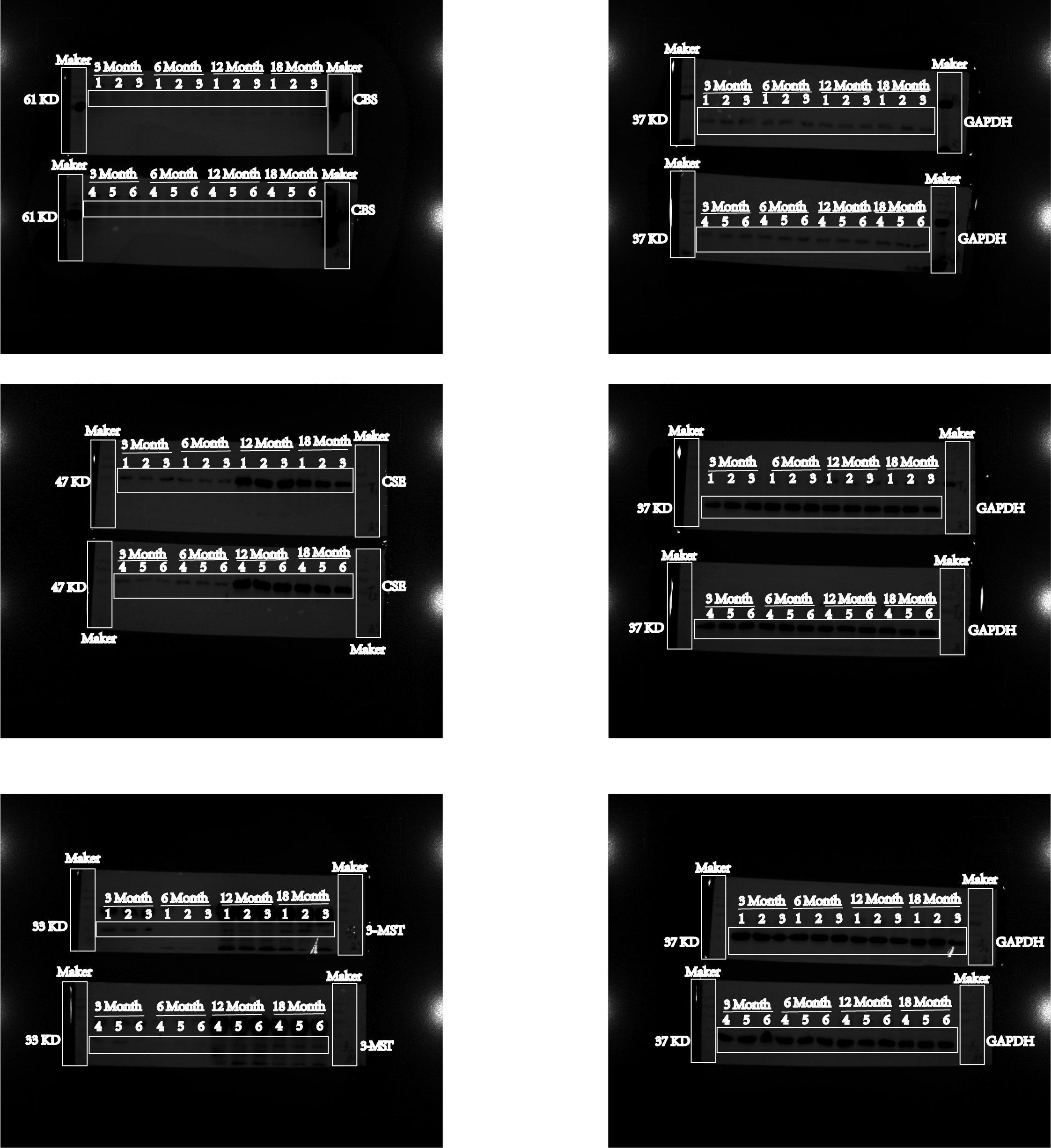

Supplement: Supplementary Figures S1-S5 [file BSR-2024-0320_supp.zip › BSR-2024-0320_suppo6B.tif]

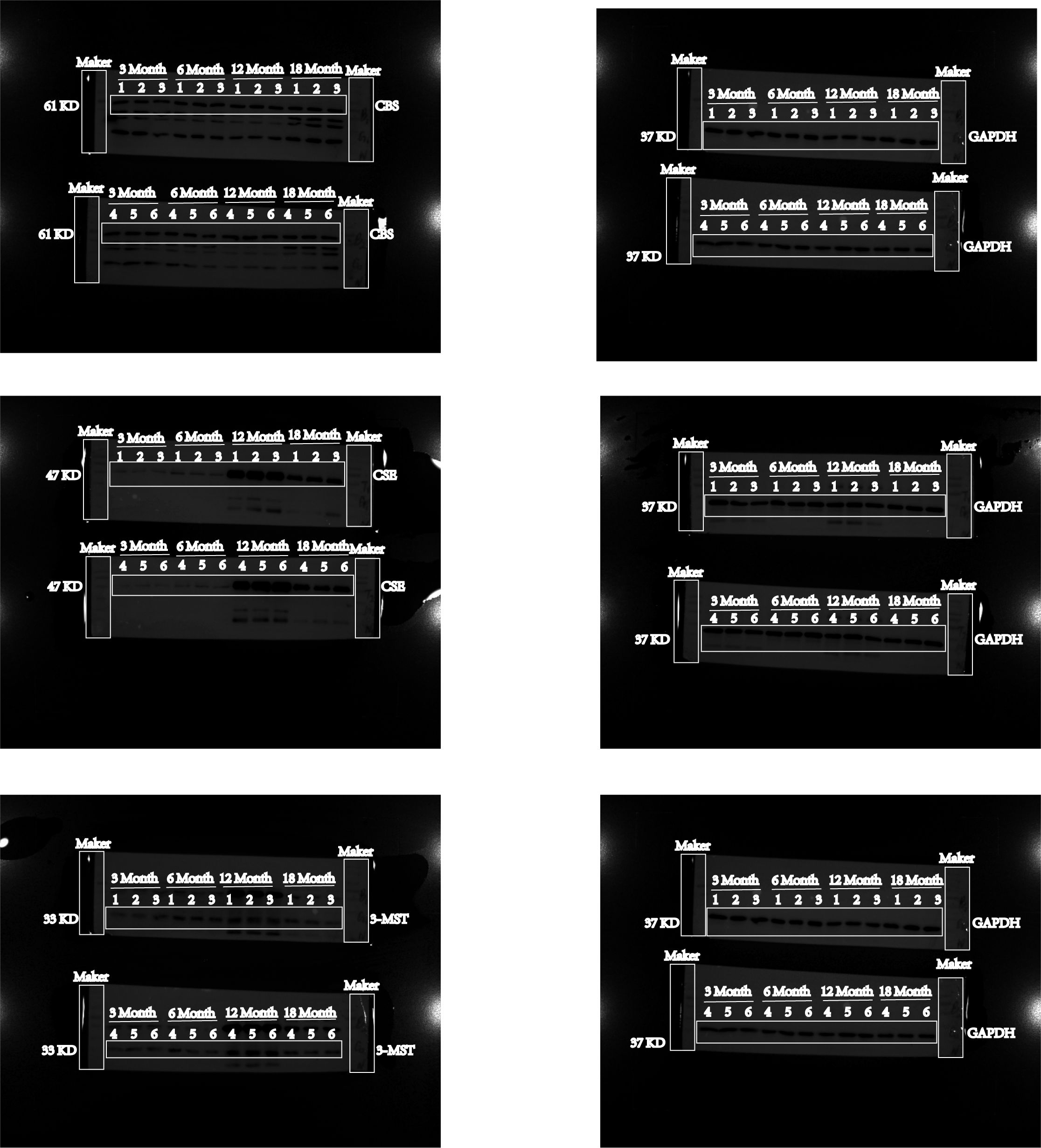

Supplement: Supplementary Figures S1-S5 [file BSR-2024-0320_supp.zip › BSR-2024-0320_suppo7B.tif]

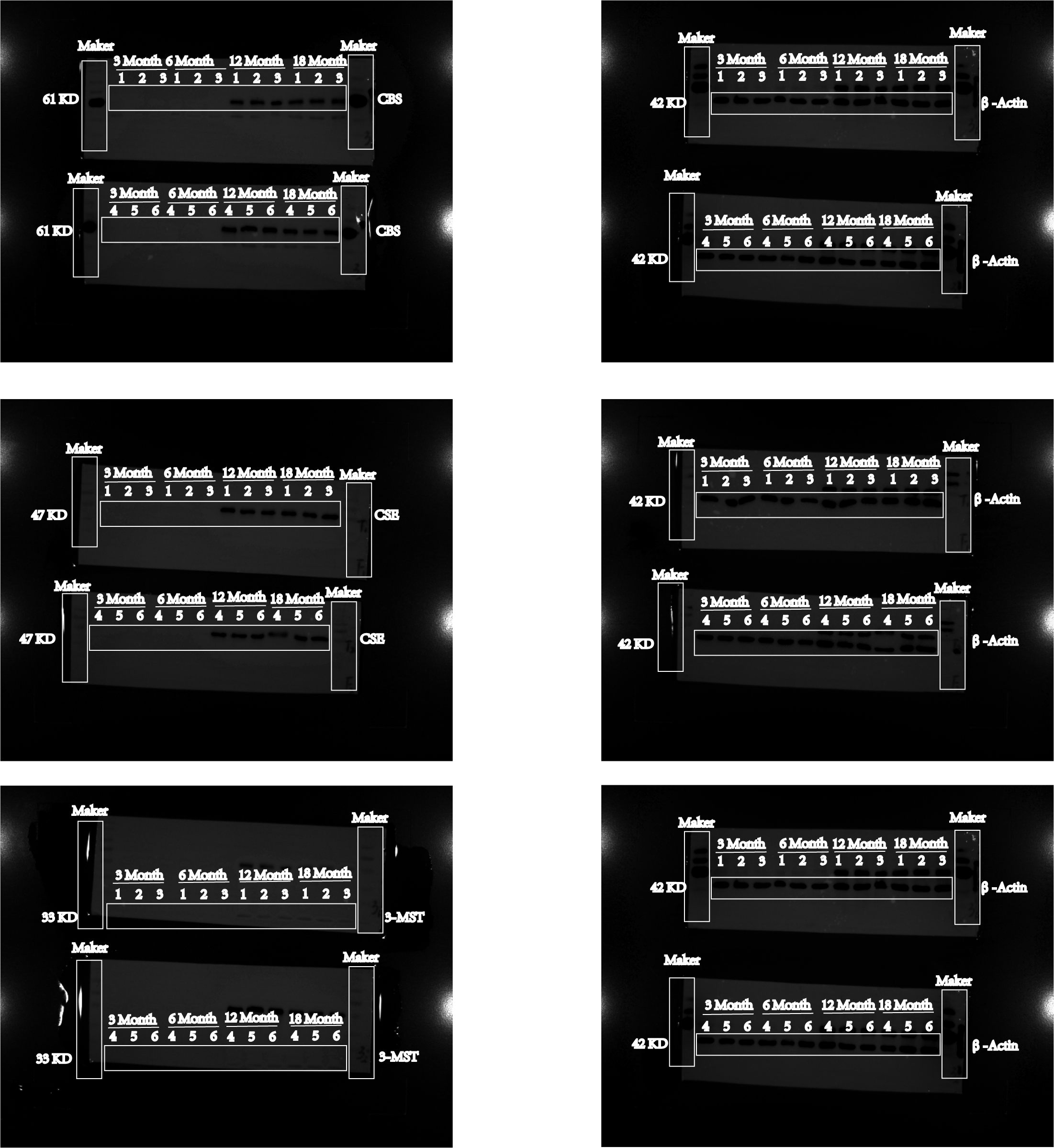

Supplement: Supplementary Figures S1-S5 [file BSR-2024-0320_supp.zip › BSR-2024-0320_suppo8B.tif]

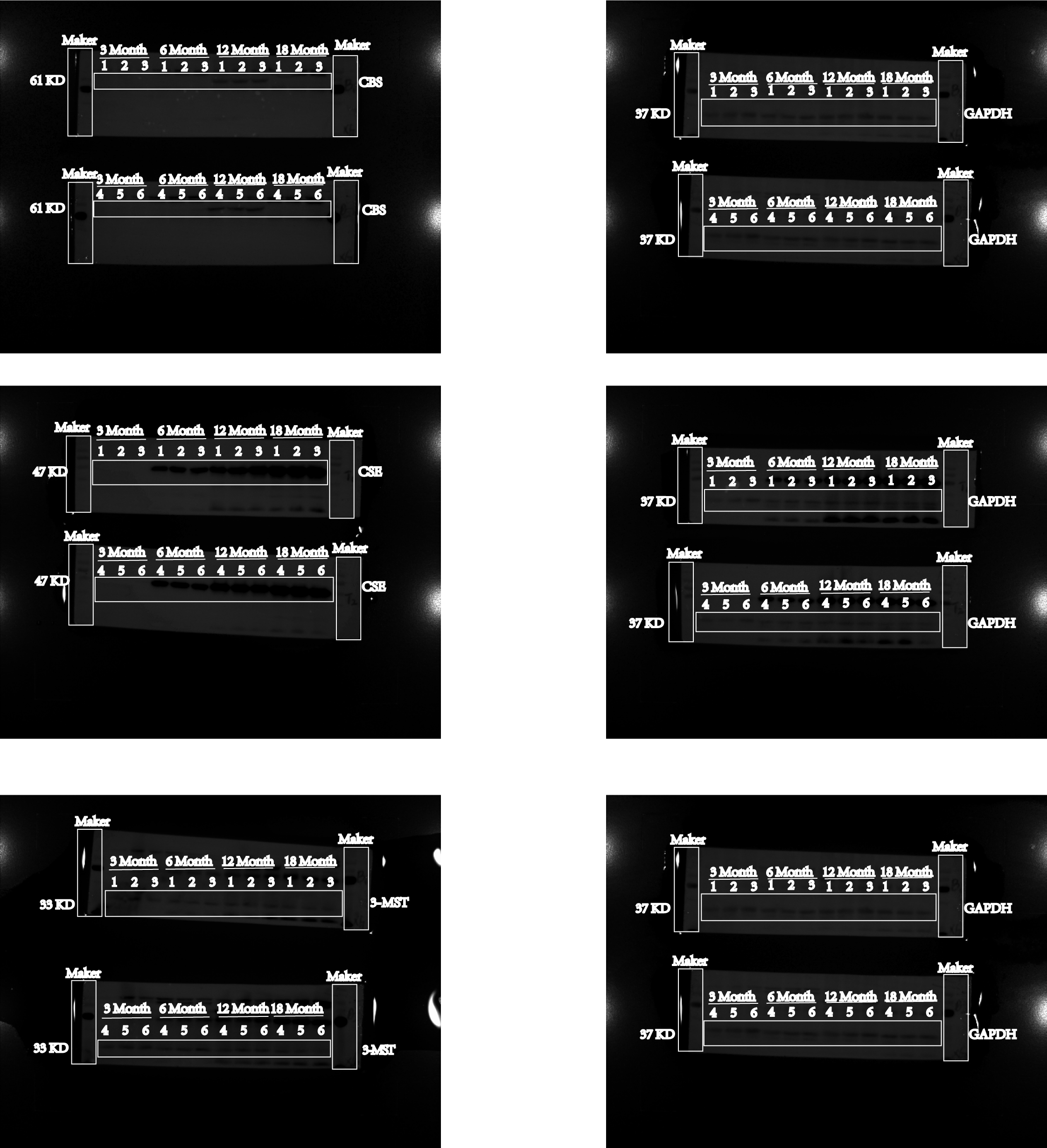

Supplement: Supplementary Figures S1-S5 [file BSR-2024-0320_supp.zip › BSR-2024-0320_suppos1B.tif]

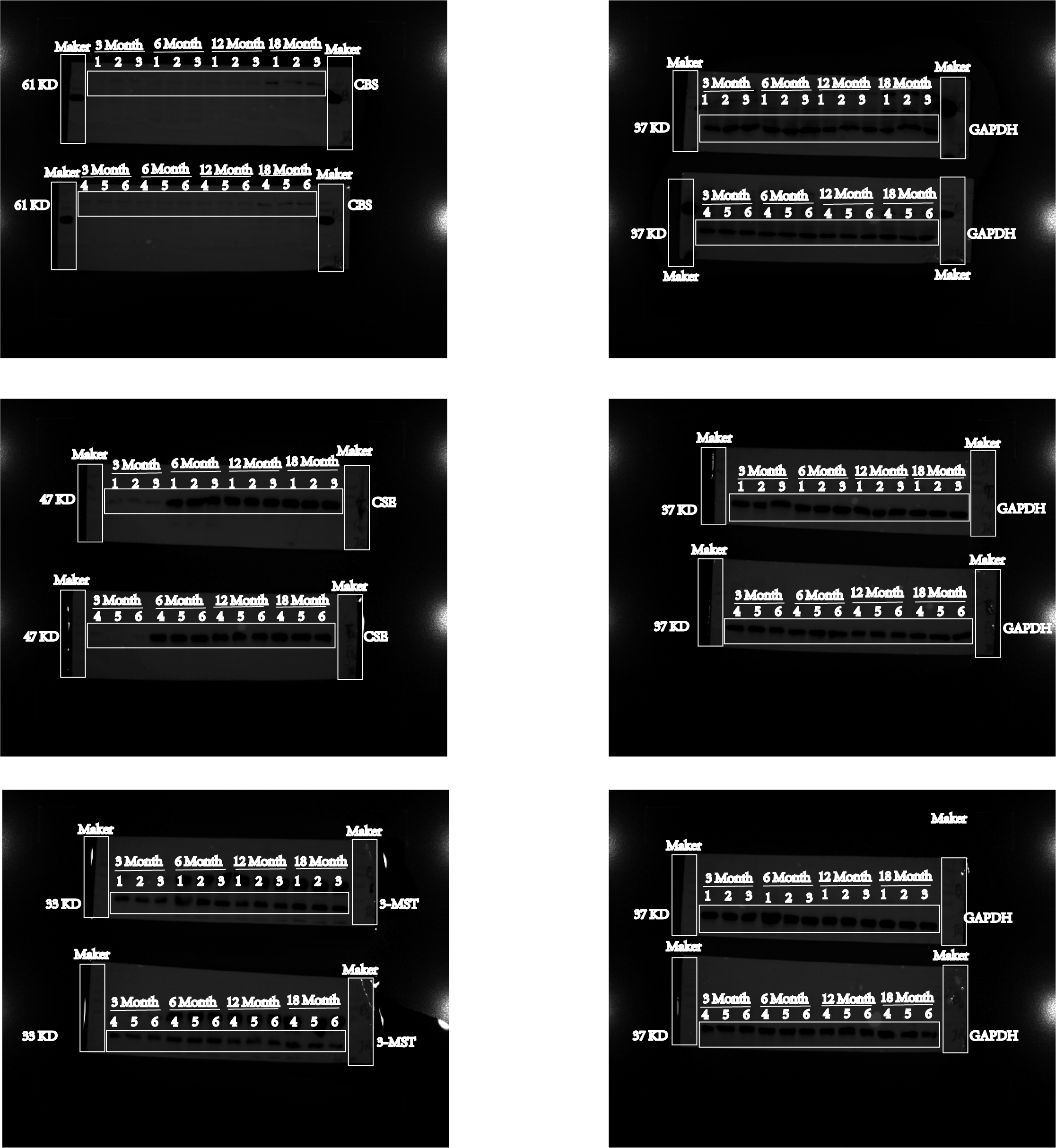

Supplement: Supplementary Figures S1-S5 [file BSR-2024-0320_supp.zip › BSR-2024-0320_suppos2B.tif]

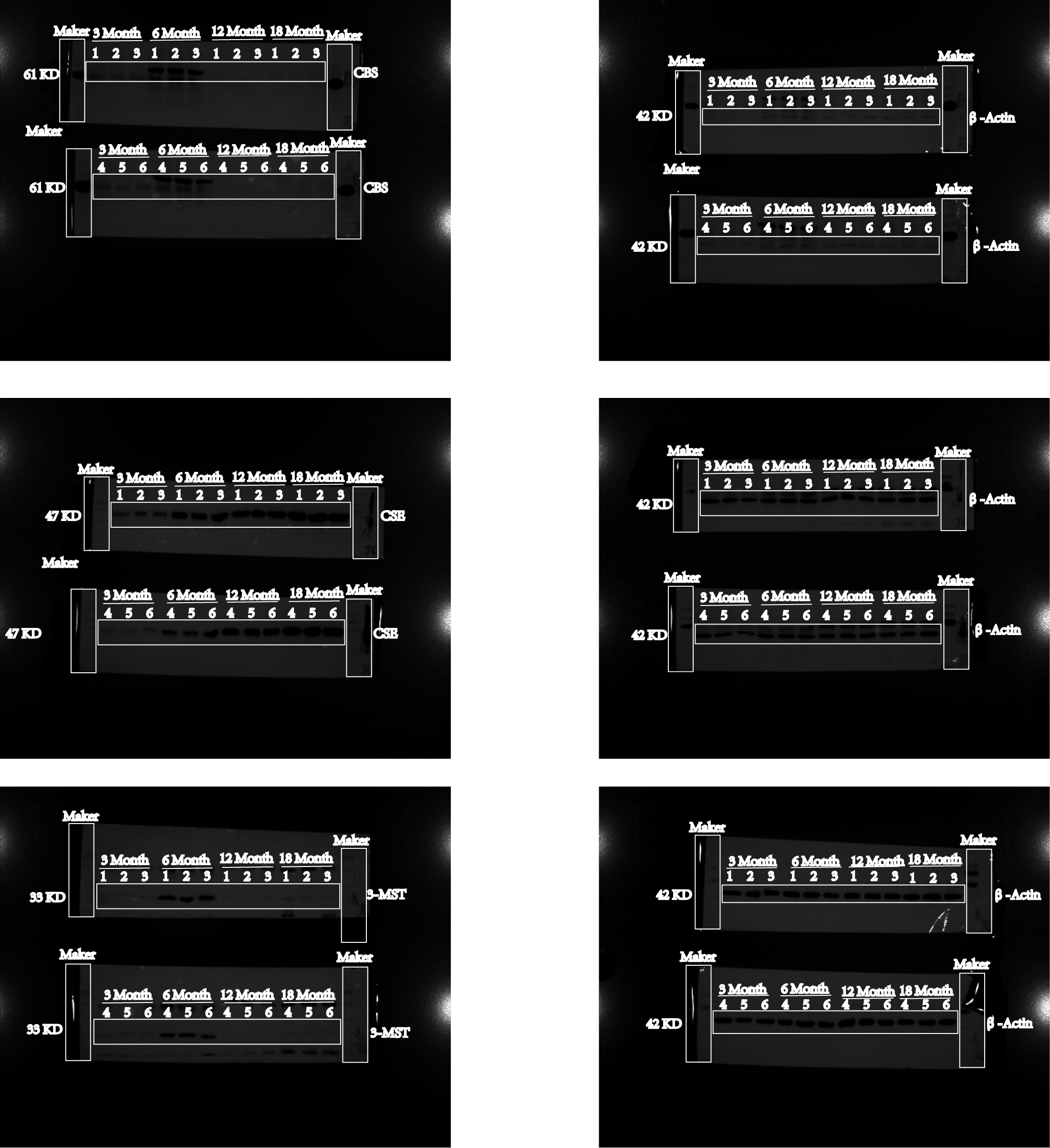

Supplement: Supplementary Figures S1-S5 [file BSR-2024-0320_supp.zip › BSR-2024-0320_suppos3B.tif]

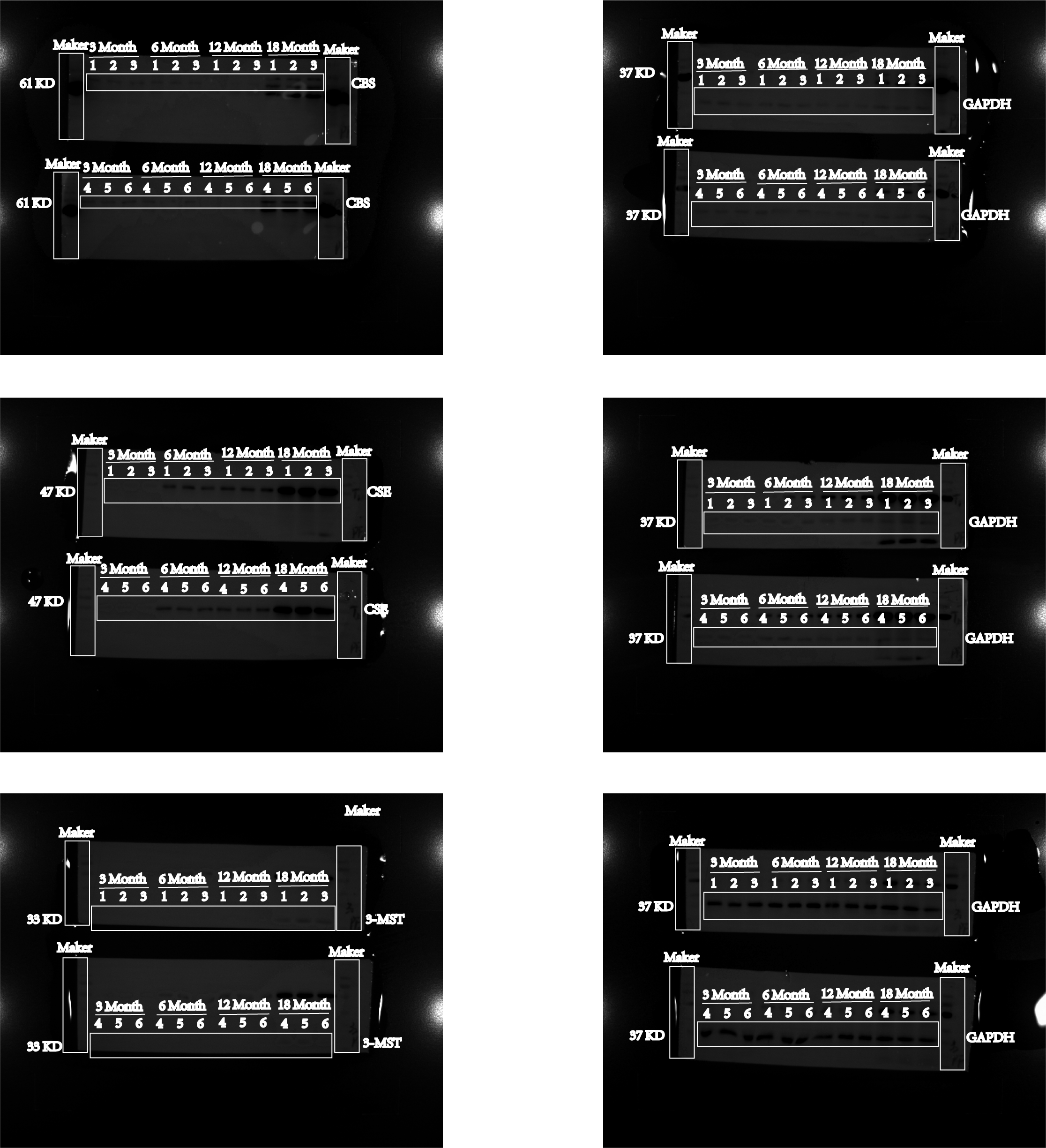

Supplement: Supplementary Figures S1-S5 [file BSR-2024-0320_supp.zip › BSR-2024-0320_suppos4B.tif]

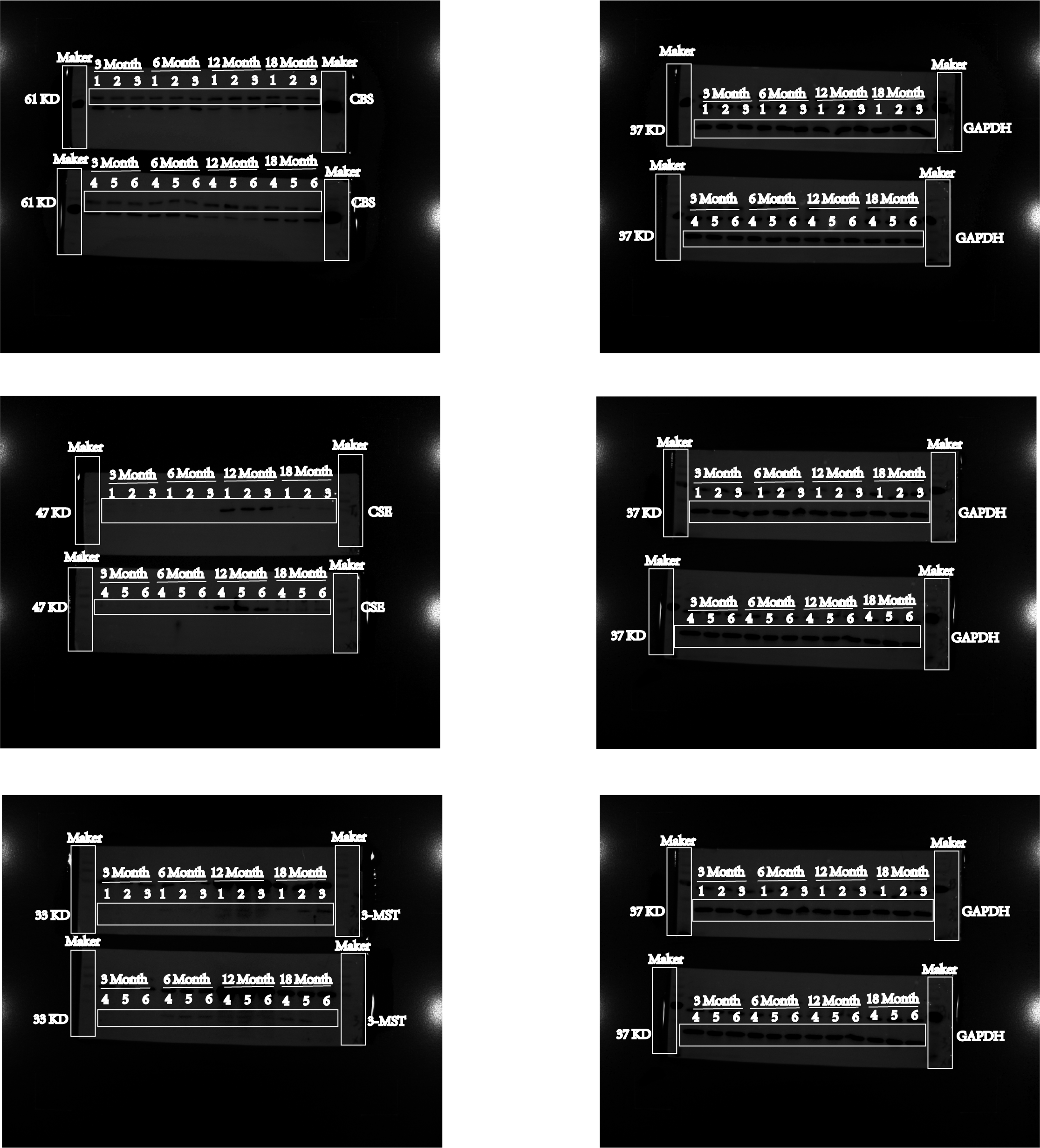

Supplement: Supplementary Figures S1-S5 [file BSR-2024-0320_supp.zip › BSR-2024-0320_suppos5B.tif]

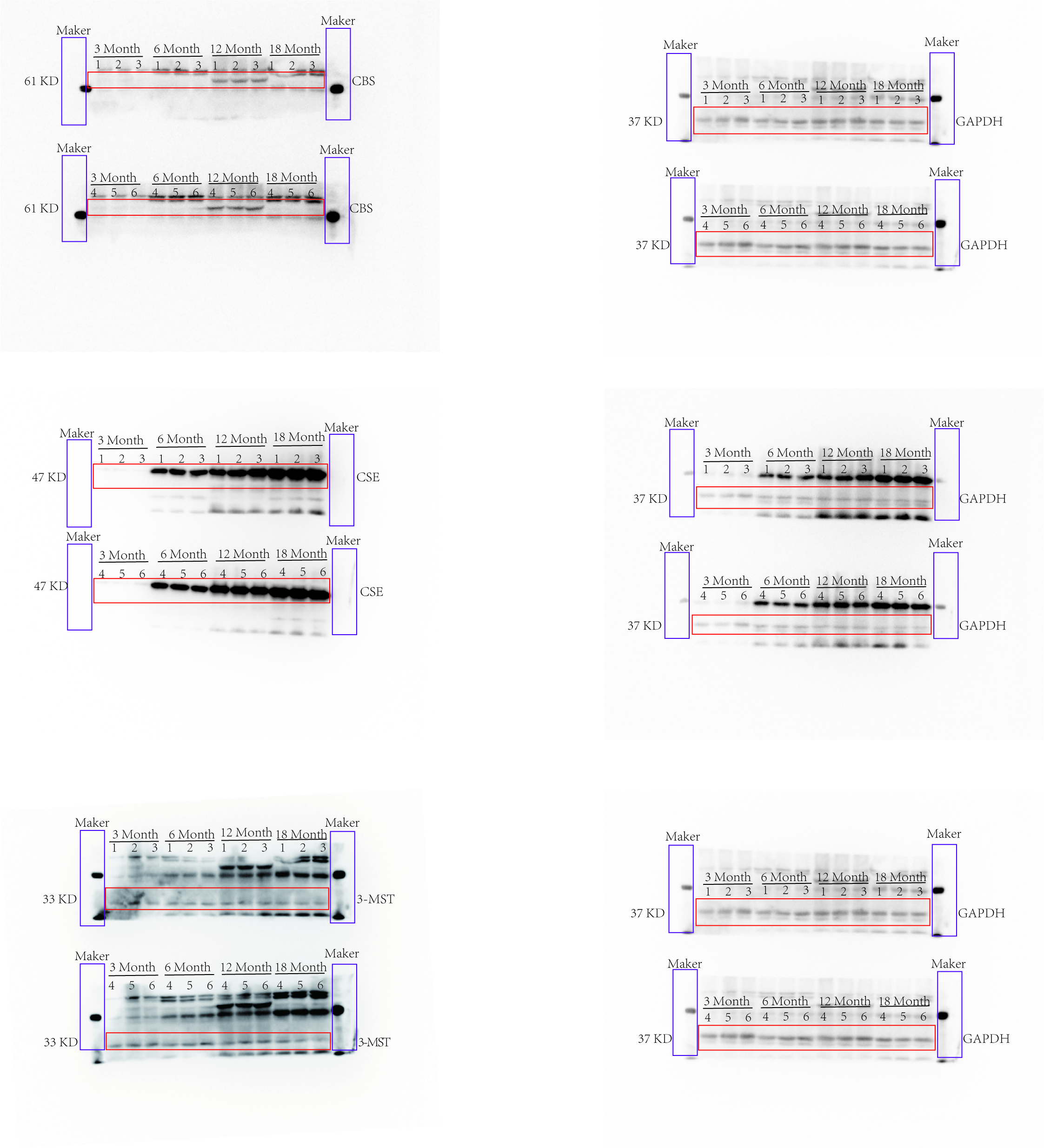

Supplement: Supplementary Figures S1-S5 [file BSR-2024-0320_supp.zip › BSR-2024-0320_supps1B.tif]

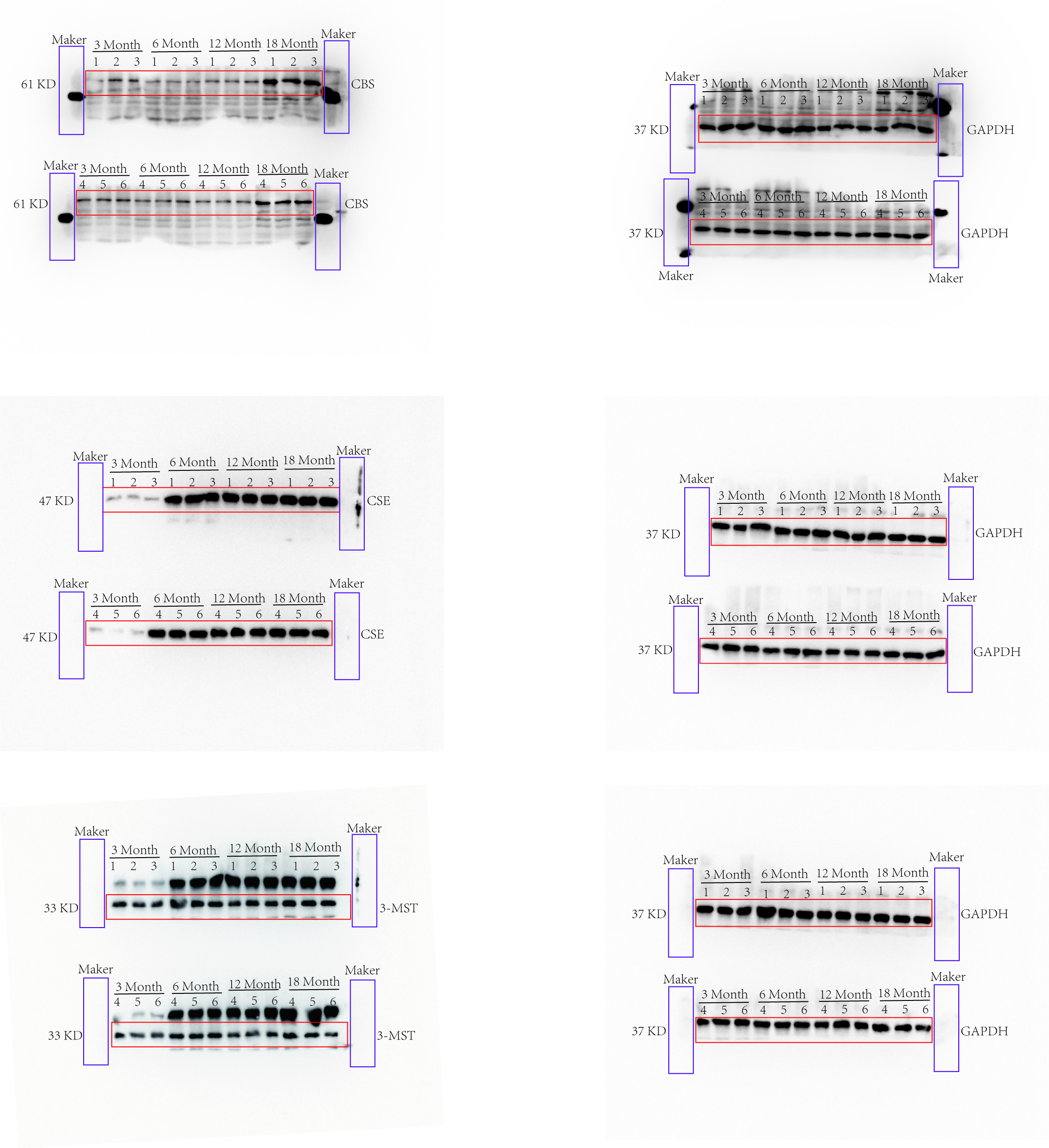

Supplement: Supplementary Figures S1-S5 [file BSR-2024-0320_supp.zip › BSR-2024-0320_supps2B.tif]

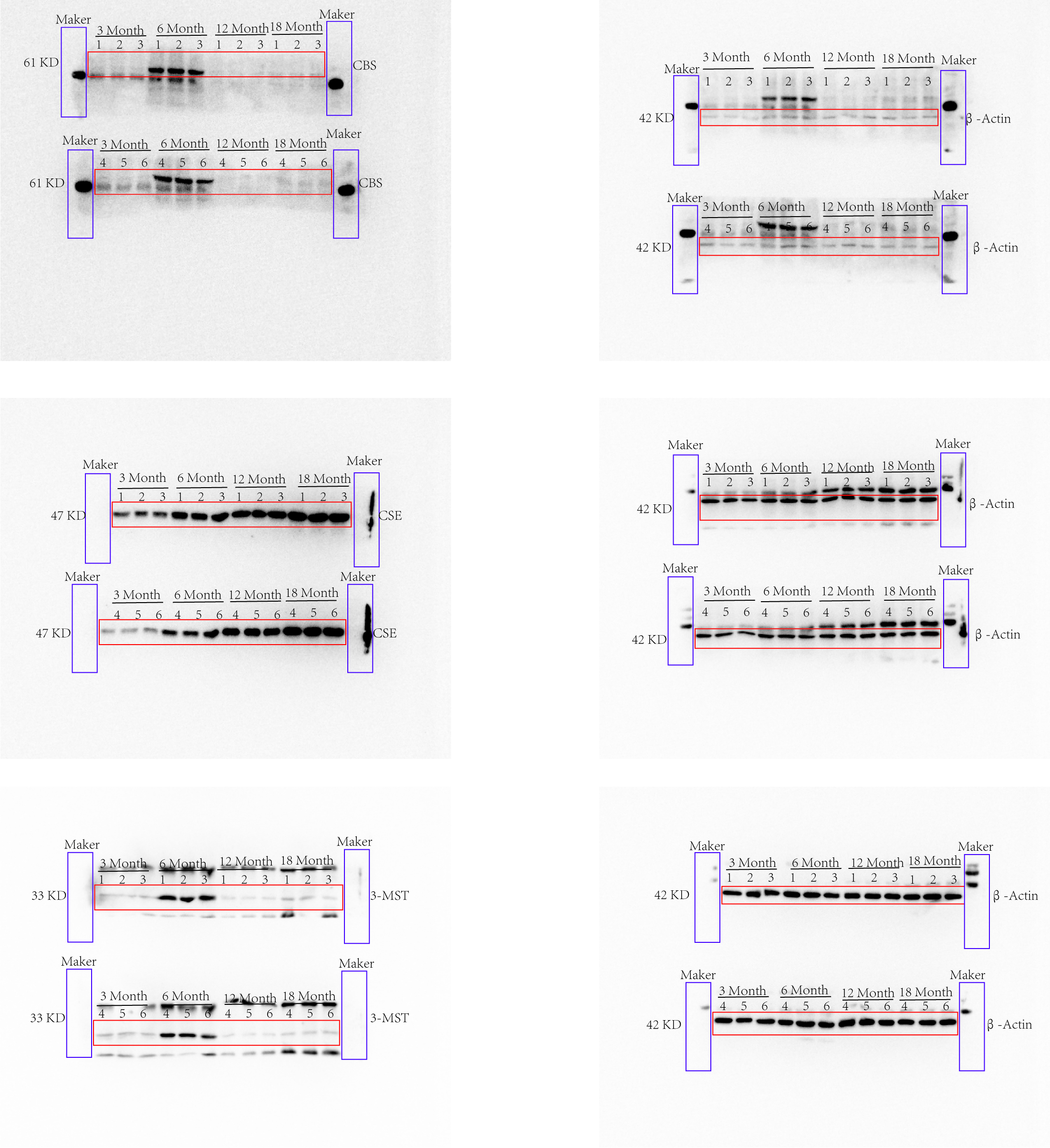

Supplement: Supplementary Figures S1-S5 [file BSR-2024-0320_supp.zip › BSR-2024-0320_supps3B.tif]

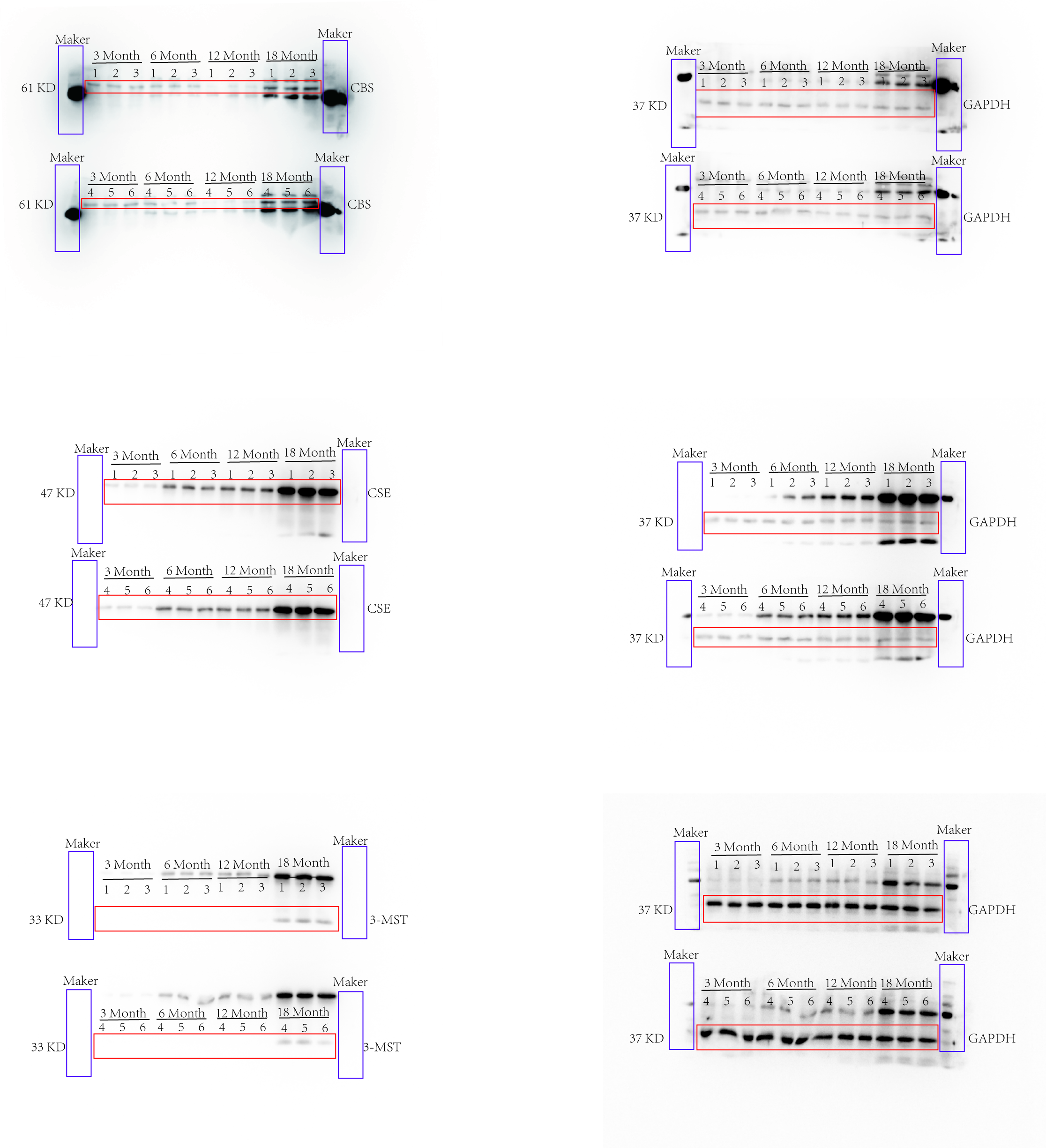

Supplement: Supplementary Figures S1-S5 [file BSR-2024-0320_supp.zip › BSR-2024-0320_supps4B.tif]

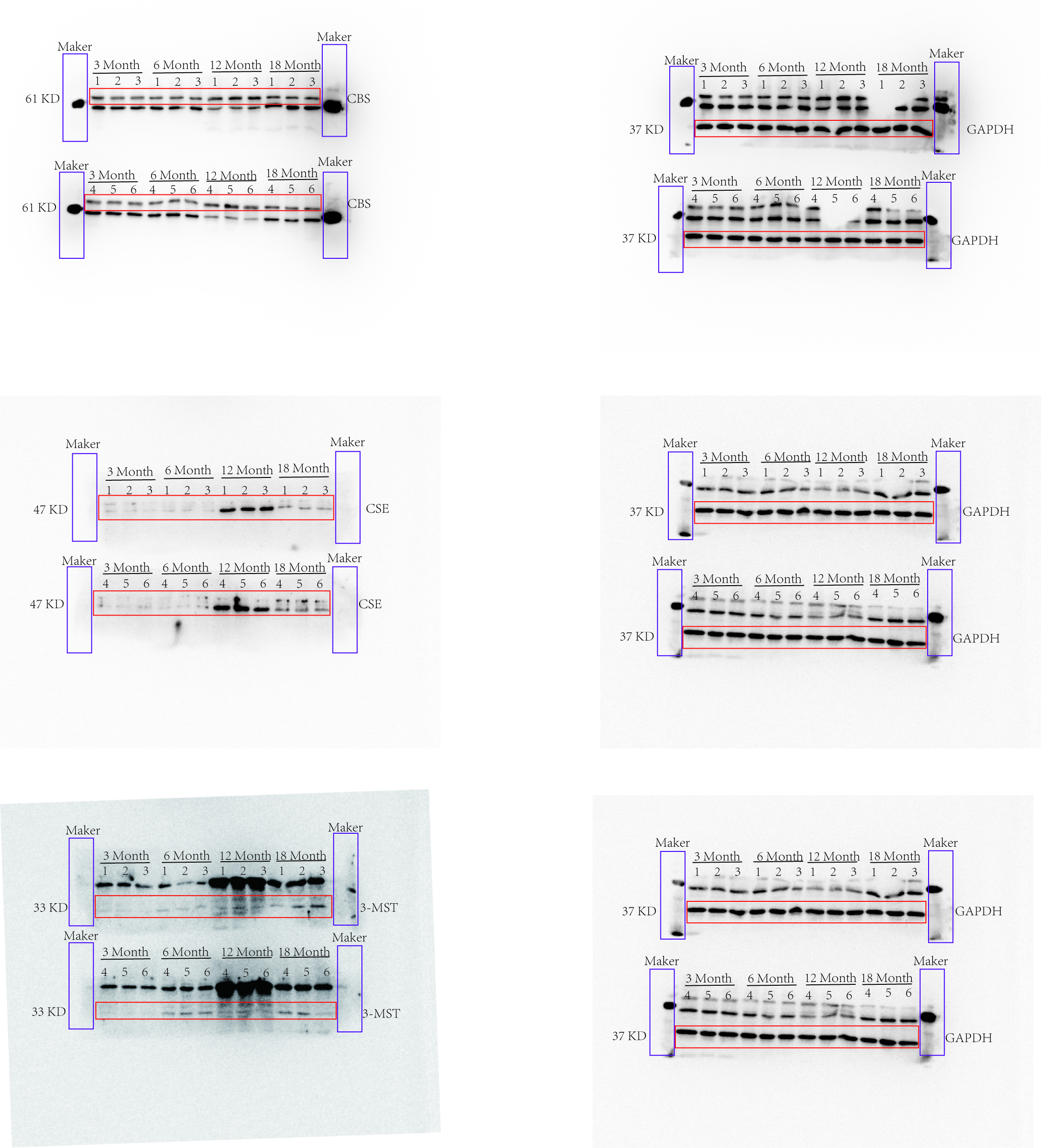

Supplement: Supplementary Figures S1-S5 [file BSR-2024-0320_supp.zip › BSR-2024-0320_supps5B.tif]
